# The Influence of Second-Order Effects on the Critical Load Multiplier and Natural Frequencies of a Low-Rise Steel Dome

**DOI:** 10.3390/ma19163445

**Published:** 2026-08-14

**Authors:** Paweł Zabojszcza, Paulina Obara, Urszula Radoń

**Affiliations:** Faculty of Civil Engineering and Architecture, Kielce University of Technology, al. Tysiąclecia Państwa Poskiego 7, 25-314 Kielce, Poland; pawelzab@tu.kielce.pl (P.Z.); paula@tu.kielce.pl (P.O.)

**Keywords:** steel dome, buckling analysis, modal analysis, geometric nonlinearity

## Abstract

This study investigates the influence of loading pattern, analysis level, and nodal connection stiffness on the stability and dynamic behaviour of a low-rise steel dome. Three joint configurations and two loading scenarios, symmetrical and asymmetrical, were analysed. Structural stability was evaluated using linear buckling analysis, second-order analysis, full geometrically nonlinear analysis. Dynamic properties were determined by modal analyses performed with and without the geometric stiffness matrix. The results show that the structural response is governed primarily by the asymmetric distribution of axial forces. For the model with pinned joints, a 68.4% decrease in the critical load multiplier is observed. Modal analysis revealed that asymmetric loading not only reduced the first natural frequency but also fundamentally changed the modal structure. Global vibration modes disappeared, modal mass became distributed over a large number of modes, and the Modal Assurance Criterion remained below 0.11, confirming a qualitative change in the vibration mechanism. In contrast, symmetrical loading caused only minor frequency reductions while largely preserving the mode shapes. The results demonstrate that linear analyses may significantly overestimate the stability and dynamic performance of low-rise steel domes under asymmetric loading. Accurate assessment therefore requires second-order effects, geometric nonlinearity, and geometric stiffness to be considered.

## 1. Introduction

Spatial bar structures made of steel are often used as supporting systems for structures with large spans, including industrial halls, commercial pavilions, station concourses, sports facilities, etc. They enable the construction of structures that require a large space between supports. Structures of this type can take various architectural and geometric forms. The axes of the members from which they are modeled form a specific spatial geometric grid. The nodes of these grids are often arranged regularly on an arbitrary base surface, i.e., a sphere, ellipsoid, paraboloid, barrel vault, etc. This surface must be selected in a way that ensures sufficient stiffness and stability within the given constraints and dominant loads. 

A special group of spatial structures are truss domes, the most common types of which are the Schwedler, Lamella, ribbed, and geodesic domes [1]. Truss domes are often cyclically symmetric structures with many elements of identical geometry, making them suitable for modern production and assembly processes. 

The behavior of domes is influenced by their geometry, material properties, and the design of the node connections. Computational models typically consider two extreme cases: pinned connections, which allow elements to rotate freely at the nodes, and rigid connections, which ensure rotational continuity between members. The idealization of connections as either fully hinged or fully rigid can lead to different results in static and dynamic analyses, especially in structures with a high degree of geometric regularity [2]. The rotational stiffness of nodes affects the global stiffness of the structure, the distribution of internal forces, the load-bearing capacity, and the structure’s resistance to loss of stability. The importance of proper joint modeling is most often felt in dynamic and structural stability analyses. In the case of lightweight spatial domes, this effect may be comparable to the effect of changes in member cross-sections or modifications to the structure’s geometry [3,4]. The inclusion of pinned joints typically leads to a reduction in the structure’s stiffness and, consequently, to a decrease in critical loads and natural frequencies. Conversely, increasing the stiffness of connections can result in higher load-bearing capacity and improved dynamic parameters, but simultaneously alters the operating mechanism of the entire system [5,6,7]. Examples of hinged connections include the SBP-1 and Mero types, while rigid connections include the WABI-1, Mero Plus, and Octaplate [8,9,10]. The behavior of the structure is also influenced by effects related to the interaction of axial forces with the deformed geometry of the system, i.e., so-called second-order effects. In structures with large spans and relatively low stiffness, such effects can lead to a significant reduction in effective geometric stiffness. Ultimately, this can affect both the values of critical load multipliers and dynamic characteristics. Structural stability assessments are often based on these values.

It is also worth citing the work [11] that analyzes the impact of ball joint degradation on the critical coefficient of single-layer domes. The authors demonstrate how joint stiffness affects the global stability of the structure. A very recent publication [12] deals with the influence of real geometric imperfections on the load-bearing capacity and buckling of mesh domes. The authors used 3D scanning data and geometrically nonlinear analysis. In [13], the authors propose a new method for modeling geometric imperfections consistent with actual manufacturing deviations.

For steel truss structures, numerical analyses accounting for imperfections and nonlinearities are a frequent subject of research. In [14], geometrically and materially nonlinear analyses were performed for various types of grid domes. Buckling studies of the elements were conducted based on the P-δ_end_ and P-δ_mid_ element test curves. Based on the element buckling results, relationships were established between element buckling and the global instability of the structure. The authors identified two instability patterns (progressive and synchronous) for typical mesh domes. In [15,16], experimental and numerical studies of truss structures were conducted. In [15], the influence of joint stiffness on the mechanical properties of barrel-shaped trusses was investigated. A good correlation was observed between experimental results and numerical models accounting for semi-rigid connections as well as material and geometric nonlinearities. Reference [16] verifies the behavior of a single-layer square-based roof structure. In the experimental analyses, the maximum load of the entire structure was determined by tracing the critical equilibrium path until failure. The results were verified against numerical analyses, yielding good correlation. The natural frequencies of the structure were also determined numerically and experimentally and compared. 

In the case of steel truss structures, modal analysis is often used to evaluate their behavior under dynamic loads, such as wind, vibrations, or seismic loads. Due to the high slenderness and relatively low spatial stiffness of such structures, determining the natural frequencies and modes of vibration is a crucial step in structural analysis. In [17,18,19,20], the authors analyze the behavior of steel domes and tensegrity structures under dynamic loads. In [21], based on experimental studies and numerical analyses, criteria for dynamic damage to steel truss structures are presented. Li et al. in [22] focused on identifying damage mechanisms in single-layer spherical truss structures. They presented valuable design recommendations for structures subjected to impact loads.

A literature review indicates that the vast majority of existing studies focus on analyzing single issues, such as the impact of geometric imperfections, linear buckling analysis, or dynamic properties. Studies that simultaneously analyze the interaction of several factors influencing dome performance are much less common. In particular, there is a lack of comprehensive analyses that allow for determining the impact of loading patterns, axial forces, the sophistication of the computational model, and the method of modeling nodal connections on the dynamic characteristics and stability of the structure. This research gap particularly concerns low-rise bar domes, whose structural response is extremely sensitive to small changes in stiffness and internal force distribution.

The aim of this study is to determine the impact of the adopted model assumptions on the safety assessment of a low-rise bar dome and to identify the factors that determine changes in its dynamic and stability properties. A multivariate approach was adopted in this study, enabling the analysis of the interaction of individual parameters of the numerical model. This allows for the identification of simplifications used in design practice that may lead to underestimation or overestimation of the structure’s safety. Analyses were conducted for two fundamentally different loading scenarios. The first corresponds to a classic load uniformly distributed over the entire dome surface and is the basic case analyzed in design standards and most scientific studies. The second scenario involves a load acting only on half the dome surface, leading to an asymmetric distribution of internal forces and the development of more complex deformations. This loading scenario corresponds to real-world operating situations, such as uneven snow accumulation, local wind action, or partial loading of the structure during assembly. Comparing the two cases allows for an assessment of the impact of load symmetry on the spatial stiffness and resistance to structural instability.

An important element of the paper is modal analysis, conducted in two variants. In the first, natural frequencies are determined ignoring the influence of axial forces, while in the second, the actual level of axial forces is taken into account. This approach allows for the assessment of the influence of compressive and tensile stresses on the effective stiffness of the structure and on changes in the natural frequencies.

The next stage of the research is a stability analysis conducted using three levels of numerical model sophistication. In the first stage, a classic linear analysis was performed, assuming the structure’s geometry remains unchanged. Next, the second-order P-Δ effect was considered. The final stage consists of a full geometrically nonlinear analysis, including large displacements and changes in the structure’s geometry during the loading process. Gradually increasing the model’s complexity allows for a quantitative assessment of the impact of nonlinear effects on the critical coefficient values and determining the applicability of simplified linear models.

Particular attention was paid to the impact of nodal connection modeling on the structure’s behavior. Three joint models were considered: a model with fully pinned connections between the lattice members and the bracing elements; a model with pinned connections between the lattice members and rigid connections between the lattice members; and a model with fully rigid connections between all members. This approach allows for the reproduction of both classical design assumptions and solutions found in real steel structures. The analysis allows for the assessment of the effect of joint stiffness on the distribution of internal forces, buckling modes, natural frequencies, and critical load multipliers.

The innovative nature of this research stems primarily from the comprehensive integration of modal and stability analyses into a single, coherent research model. This broad scope of research enables the identification of interdependencies between the analyzed factors and the assessment of their combined impact on structural safety. Unlike most available studies, individual aspects are not analyzed independently, but as elements of a single, interacting system.

A significant achievement of this work is also the quantitative assessment of the impact of subsequent simplifications of the numerical model on the obtained results. This allows us to determine which model elements have a dominant influence on the safety assessment and to identify situations in which the use of simplified linear analyses may lead to incorrect interpretation of the structure’s behavior.

The conducted analyses have both cognitive and applied significance. From a scientific perspective, they expand knowledge of the interrelationships between the dynamic properties, stress state, and stability of low-rise bar domes. From a practical perspective, they provide designers with information enabling the rational selection of numerical models and the assessment of the impact of the adopted simplifications on structural safety. The results can be a basis for formulating recommendations for the modeling of three-dimensional bar structures, especially in cases of asymmetric loads and analyses requiring consideration of second-order effects and geometric nonlinearity.

## 2. Materials and Methods

### 2.1. Theoretical Background: Buckling and Modal Analysis

In the design of steel structures, one of the fundamental issues is ensuring an adequate level of safety for load-bearing members. The verification of structures conducted in accordance with the requirements of PN-EN 1993-1-1 [23] includes checking the load-bearing capacity of cross-sections and assessing the stability of members and entire structural systems. A particularly important issue is the analysis of the overall stability of members subjected to compression and compression-bending, for which loss of stability may occur before the material’s ultimate strength is reached. A correct safety assessment requires the use of an appropriate calculation model that accounts for the actual mode of operation of the structure, including support conditions, joint stiffness, and the influence of deformation on the distribution of internal forces [24,25].

Loss of global stability in steel members may take the form of flexural, torsional, or flexural-torsional buckling, while in members subjected to bending, warping may occur. The susceptibility of members to these phenomena increases with an increase in their slenderness and a decrease in the stiffness of the structure.

In design practice, various computational models are used, the selection of which depends on the anticipated behavior of the structure and the degree of influence of deformation on its performance. In the design of steel structures, a distinction is made between first-order analysis and second-order analysis. In first-order linear analysis (LA), it is assumed that the structure’s geometry remains unchanged under load and that there is a linear relationship between stresses and strains. This method uses the principle of superposition and can be applied only when the effect of deformation on the distribution of internal forces is negligible [26]. 

The sensitivity of the structure to second-order effects, in accordance with the requirements of PN-EN 1993-1-1 [23], is assessed, among other things, based on the critical load multiplier α_cr_ obtained from a linear buckling analysis (LBA):

(1) $$[K_{L}\;+\;\alpha \cdot K_{G}]\cdot q\;=\;0$$

where: **K_L_**—linear stiffness matrix, **K_G_**—geometric stiffness matrix, α—eigenvalues, **q**—vector of nodal displacements.

The parameter α defines the relationship between the critical load and the design load and serves as a measure of the structure’s stability margin. In accordance with the recommendations of standard PN-EN 1993-1-1 [23], the influence of second-order effects can be considered negligible if the critical coefficient is sufficiently high. For typical elastic global analyses, second-order effects may be neglected when α_cr_ ≥ 10 [23]. It should be emphasized, however, that the α_cr_ multiplier is merely an indicator of the structure’s overall susceptibility to loss of stability. In the case of spatial structures, especially lightweight steel domes, the actual behavior of the system may also be influenced by geometric imperfections, the stiffness of node connections, the distribution of compressive forces, and local changes in structural stiffness. For this reason, an analysis accounting for second-order effects can provide additional information even for structures that meet standard requirements. According to the provisions of the standards, if 3.0 < α_cr_ < 10.0 [23], the use of approximate second-order linear elastic analysis (P-Δ analysis) is permitted, whereas if α_cr_ < 3 [23], the use of full geometrically nonlinear analysis (GNA) is recommended. 

From a mathematical point of view, accounting for second-order effects leads to the extension of the classical system of equilibrium equations by a geometric stiffness matrix dependent on the axial forces present in the structure. In P-Δ analysis, the influence of deformation is accounted for by an additional geometric matrix corresponding to the global displacements of the system:

(2) $$(K_{L}\;+\;K_{G}(q))\;q\;=\;P$$

Since the stiffness matrix depends on the current state of internal forces and structural deformations, the system of equations is nonlinear and requires an iterative solution.

The equilibrium equation can be written as:

(3) $$K_{T}(q)\;\cdot \;\Delta q\;=\;\Delta \alpha \;\cdot \;P\;+\;R$$

where: **K_T_**—tangent stiffness matrix, Δ**q**—displacement increment, Δα—load parameter increment, **P**—external load vector, **R**—residual force vector.

The tangent stiffness matrix **K_T_** of the structure is obtained by aggregating the stiffness matrices of the individual elements $K_{T}^{e}$:

(4) $$K_{T}\;=\sum_{i=1}^{e}K_{T}^{e}=\;\sum_{i=1}^{e}\left(K_{L}^{e}\;+\;K_{\mathrm{GN}}^{e}\;+\;K_{u1}^{e}+\;K_{u2}^{e}\right)$$

where: $K_{T}^{e}$—tangent stiffness matrix of the element, $K_{L}^{e}$—linear stiffness matrix of the element, $K_{GN}^{e}$—geometric stiffness matrix of the element, $K_{u1}^{e}$ and $K_{u2}^{e}$—nonlinear stiffness matrices of the element.

This system of equations can be solved using iterative methods [27] or Riks’s fixed-arc-length method [28,29], which are typically implemented in computer programs. 

The topic of tracking and verifying equilibrium paths is frequently addressed in scientific research. In [30,31], the results of a series of experimental and numerical studies for a low-rise and high-rise truss structure are presented. Various variants of numerical simulations were compared with experimental results. It was noted that even minor local imperfections can significantly affect the performance of low-rise structures. The authors of [32] verified the sensitivity of the equilibrium path of structures that may be susceptible to loss of stability in the form of a nodal instability. 

The results obtained from LBA and GNA analyses enable the assessment of a structure’s stability and the determination of its behavior under static loads. These analyses are supplemented by modal analysis, which evaluates the structure’s dynamic properties, particularly its natural frequencies, vibration modes, and damping coefficients. Modal analysis allows for the effective resolution of structural problems related to vibrations during the structure’s operation and even during the design phase. The primary objective is to compare the excitation frequencies in the system with the structure’s natural frequencies. If these values converge or coincide, the structure is at risk of resonance, i.e., a multiple amplification of vibrations, which can lead to damage or complete destruction of the building. Natural frequencies and eigenvectors are obtained from the following formula:

(5) $$(K_{L}\;-\;\omega_{i}^{2}M)\cdot q_{i}\;=\;0$$

where: **M**—the mass matrix of the structure, ω**_i_**—the natural frequency (angular natural frequency) of the i-th vibration mode [rad/s], $f_{i}=\frac{\omega_{i}}{2\pi }-\mathrm{frequency}$ [Hz], **q_i_**—eigenvector of the i-th vibration mode.

Eigenvectors are normalized such that the largest value of each is equal to 1.0. 

Currently, the default method for solving the eigenvalue problem is the Lanczos algorithm [33,34], which is an effective approach to solving large-scale eigenvalue problems. It allows for the determination of the required n eigenvalues and eigenvectors with arbitrary precision.

The natural frequencies of a structure are influenced, among other factors, by the static forces applied to it. The determination of structural vibrations, taking static forces into account, proceeds in two stages. In the first stage, the internal forces in the members are calculated, which are necessary to determine the matrix $K_{G}\;\mathrm{or}\;K_{\mathrm{GN}}$ in the current configuration. The static load vector **P** can represent a single load case or a combination of defined load cases. In the second step, the eigenvalues and eigenvectors are determined from the following equations:

(6) $${[(K}_{L}+K_{\mathrm{GN}})\;-\;\omega_{i}^{2}M]\cdot q_{i}\;=\;0\;\;\mathrm{or}\;\;[(K_{L}+K_{G})\;-\;\omega_{i}^{2}M]\cdot q_{i}\;=\;0$$

Positive eigenvalues describe stable equilibrium states, negative eigenvalues describe unstable equilibrium states, while a zero eigenvalue indicates a stability problem (buckling). A negative determination of the matrix (**K_L_** + $K_{\mathrm{GN}}$) or (**K_L_** + $K_{G}$) indicates that the static load is approaching the critical (buckling) value.

### 2.2. Numerical Model

#### 2.2.1. Description of the Element 

**Figure 1:**
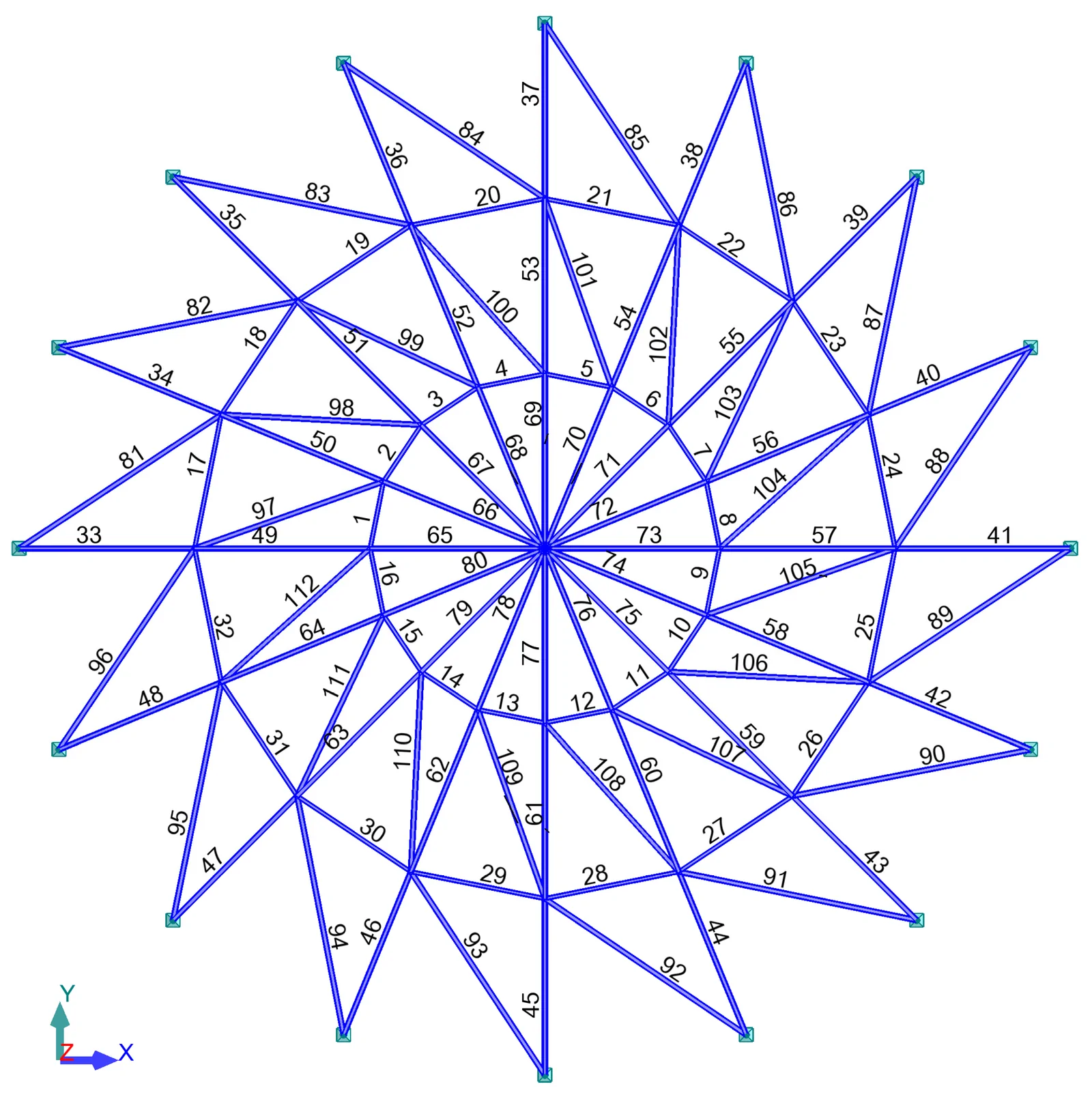
Structure geometry with member numbering.

In the paper, a single-layer steel dome was analyzed. The structural analysis was performed using Autodesk Robot Structural Analysis 2023 software. The dome consists of 49 nodes and 112 members. The geometry of the dome is presented in Table A1 (Appendix A) and Figure 1. 

The structure is divided into four groups of members: meridians (members 33–80), upper parallels—R1 (members 1–16), lower parallels—R2 (members 17–32), and bracing (members 81–112). 

**Figure 2:**
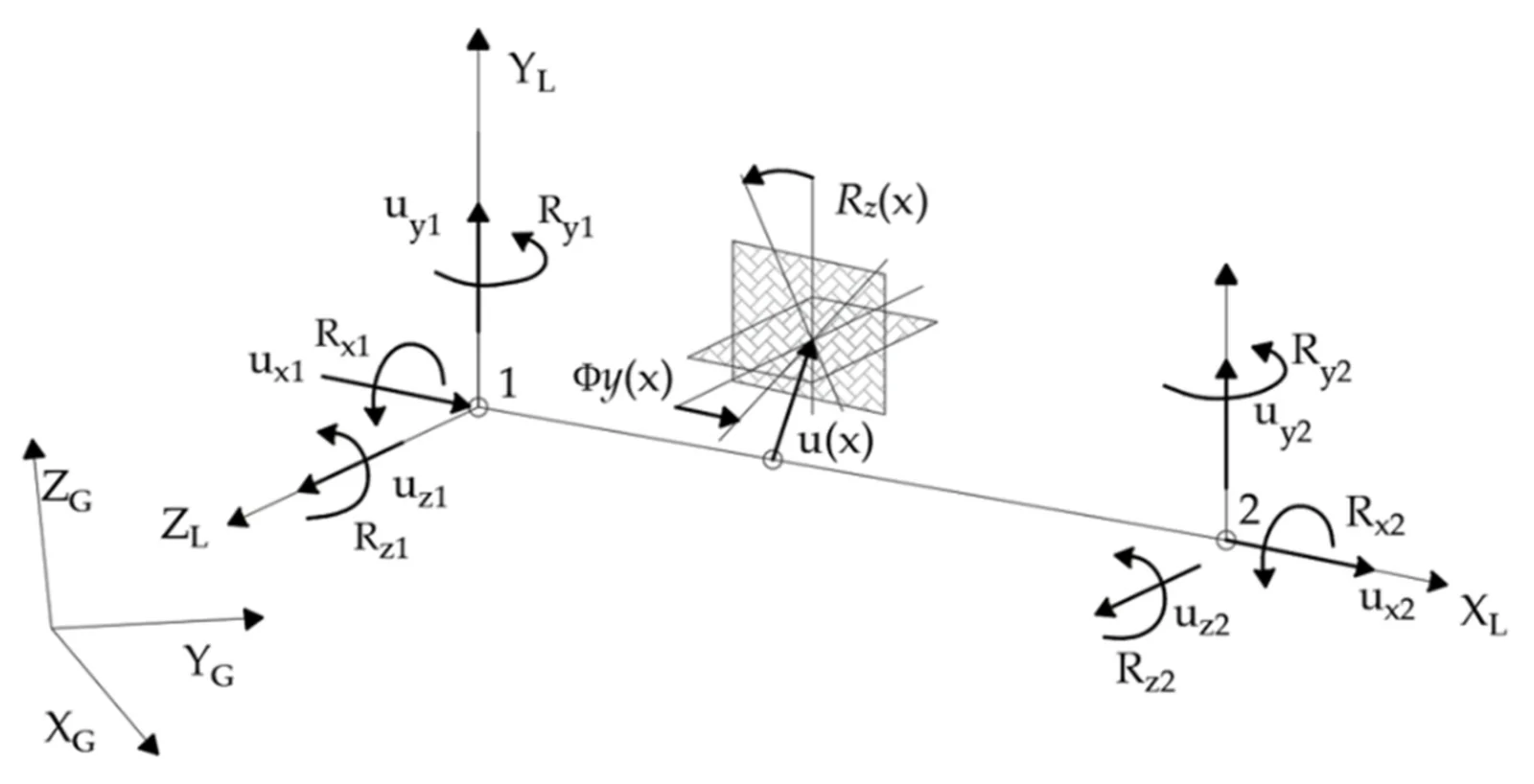
Sign convention and degrees of freedom of frame element [17].

The numerical model of the analyzed bar dome was developed using three-dimensional frame elements (Figure 2). Each node of the structure was described by six degrees of freedom, comprising three linear displacement components (u_x_, u_y_, u_z_) and three rotational components (R_x_, R_y_, R_z_). Displacements were defined relative to the global coordinate system (X_G_, Y_G_, Z_G_), while rotations were related to the axes of this system. Each element had a unique local coordinate system defined as a right-handed Cartesian coordinate system. The local *x*-axis was identified with the longitudinal axis of the element and assumed a direction consistent with the orientation from the starting node to the end node. The remaining local axes, y and z, were located in the plane of the bar cross-section and determined according to the right-handed coordinate system principle. In the adopted convention, the *y*-axis corresponded to the cross-sectional axis associated with the greater moment of inertia, while the *z*-axis corresponded to the axis with the lower moment of inertia, depending on the geometry of the element’s cross-section.

Bars are divided into several finite elements to ensure an accurate representation of buckling phenomena and geometrically nonlinear structural behavior. Modeling an entire member with a single finite element may not adequately capture the curvature of the member axis during buckling, which can lead to an overestimation of its stiffness and, consequently, an overestimation of the critical buckling load. A refined finite element mesh enables a more accurate representation of buckling modes, internal force distribution, and displacements, while also providing a more reliable consideration of second-order effects and stiffness changes resulting from axial forces.

In engineering practice, compression members are typically discretized into at least 4–8 finite elements, whereas 8–20 finite elements are recommended for buckling and geometrically nonlinear analyses, particularly for slender members. The final number of finite elements should be determined through a mesh convergence study, in which the mesh is progressively refined until further refinement no longer produces significant changes in critical load multiplier, displacements, or internal forces. In the present study, the adopted discretization ensured the convergence of both the modal and buckling analyses, allowing for an accurate representation of buckling modes and the influence of geometric nonlinearity on the critical load multiplier and natural frequencies.

#### 2.2.2. Description of the Material

Square hollow sections (SHS) conforming to PN-EN 10210-2:2019-06 [35] were adopted in the numerical analyses, while the material properties were assumed for S355J2H structural steel in accordance with PN-EN 10210-1:2019-06 [36]. S355J2H is widely used in structural applications owing to its favorable mechanical properties, good weldability, and reliable structural performance. According to the standard, the steel is characterized by a minimum yield strength of 355 MPa, a tensile strength of 470–630 MPa, and a minimum elongation after fracture of 22%.

No experimental material characterization or chemical analysis was performed in the present study. Therefore, the numerical model was developed using the nominal material properties specified in PN-EN 10210-1. The limiting chemical composition of S355J2H steel according to the standard is presented in Table 1.

Silicon primarily acts as a deoxidizing element during steelmaking, whereas manganese is mainly added to control the effects of sulfur and to improve the hot-working characteristics of the steel. The contents of both elements are specified by the standard as limiting values rather than alloying additions intended to directly increase the mechanical strength. The required mechanical properties of S355J2H are achieved through the combined effect of the specified chemical composition and the thermomechanical manufacturing process, including hot rolling and controlled cooling, which produces the required microstructure.

The relationship between stress and strain was investigated within the linear elastic range. In this region, the material behavior was described using Hooke’s law, which assumes a proportional relationship between the applied stress and the resulting strain. The linear elastic model was adopted under the assumption that the material returns to its original state after the removal of external loading, provided that the yield limit is not exceeded.

For the analyzed steel components, the elastic response was defined by the Young’s modulus (E = 210 GPa) and Poisson’s ratio (ν = 0.3), which characterize the stiffness and deformation behavior of the material. The application of the linear elastic model allows for the accurate evaluation of stress and strain distributions in structural elements subjected to loads within the elastic range.

#### 2.2.3. Boundary Conditions

The analyzed structure is supported by a reinforced concrete ring beam, which serves as the foundation element for the steel dome composed of square hollow section (SHS) members. The ring beam provides a rigid and stable support condition, ensuring effective load transfer from the steel superstructure to the foundation while limiting displacements within the support zone. In the finite element method (FEM) model, hinged boundary conditions were applied at the supports, allowing the transmission of translational forces while eliminating the transfer of bending moments. This idealized support assumption enables an accurate representation of the global structural response of the spatial bar dome and facilitates the assessment of stress and strain distributions within the steel members.

#### 2.2.4. Description of the Loading

In the numerical analysis, the applied loads were defined as vertical concentrated forces directly applied to the nodes of the structure. The magnitude of each nodal load was assumed to be 30 kN. The load directions and node numbering convention were defined according to the adopted global coordinate system. To evaluate the structural response of the steel bar dome, two load cases were considered, including symmetric and asymmetric loading conditions. 

**Figure 3:**
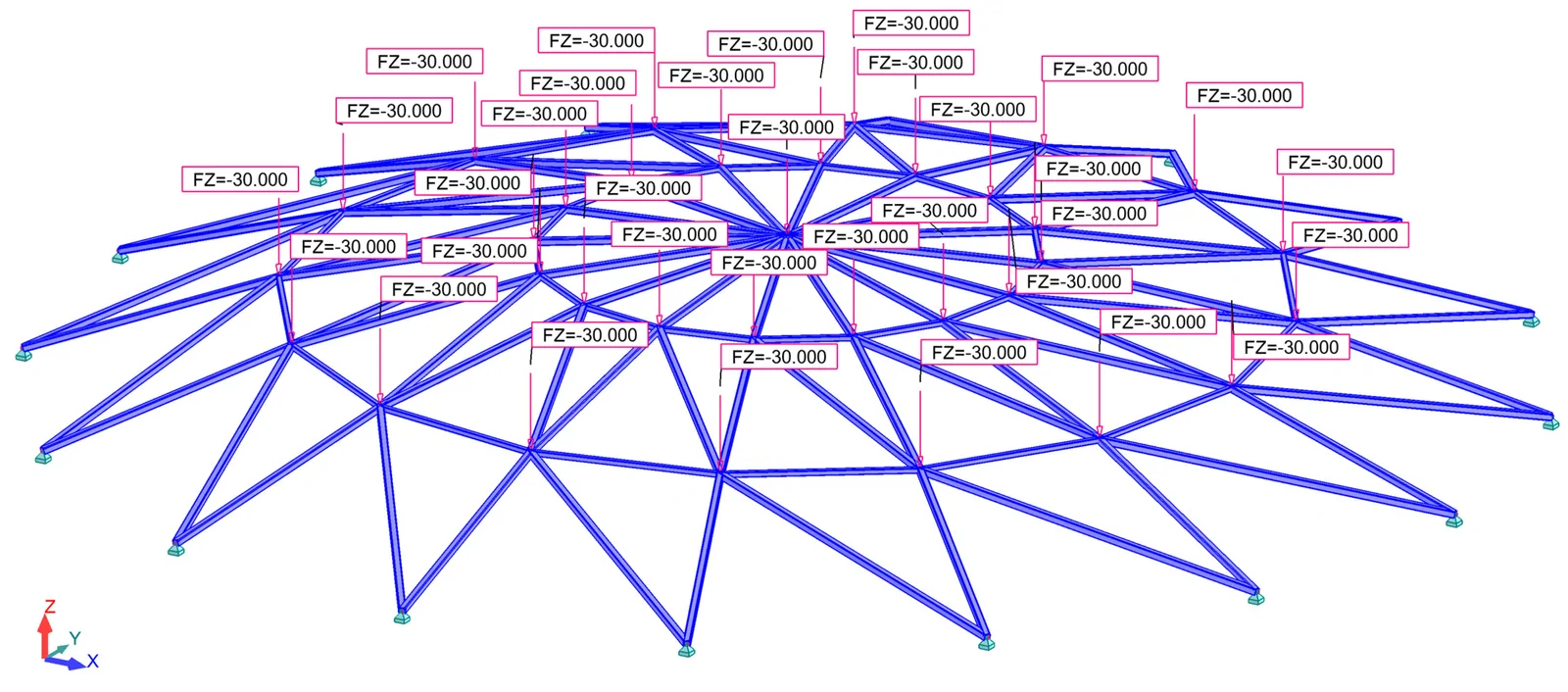
Structural symmetric load diagram [kN].

Load Case 1—Symmetric loading condition:

A symmetric nodal loading was applied over the entire surface of the dome (Figure 3). This load case represents typical loading scenarios, such as uniform snow loading or permanent loads resulting from additional structural components. Under this loading condition, the structure exhibits an approximately axisymmetric response, with the dominant load transfer mechanism being axial force transmission through the bar members.

**Figure 4:**
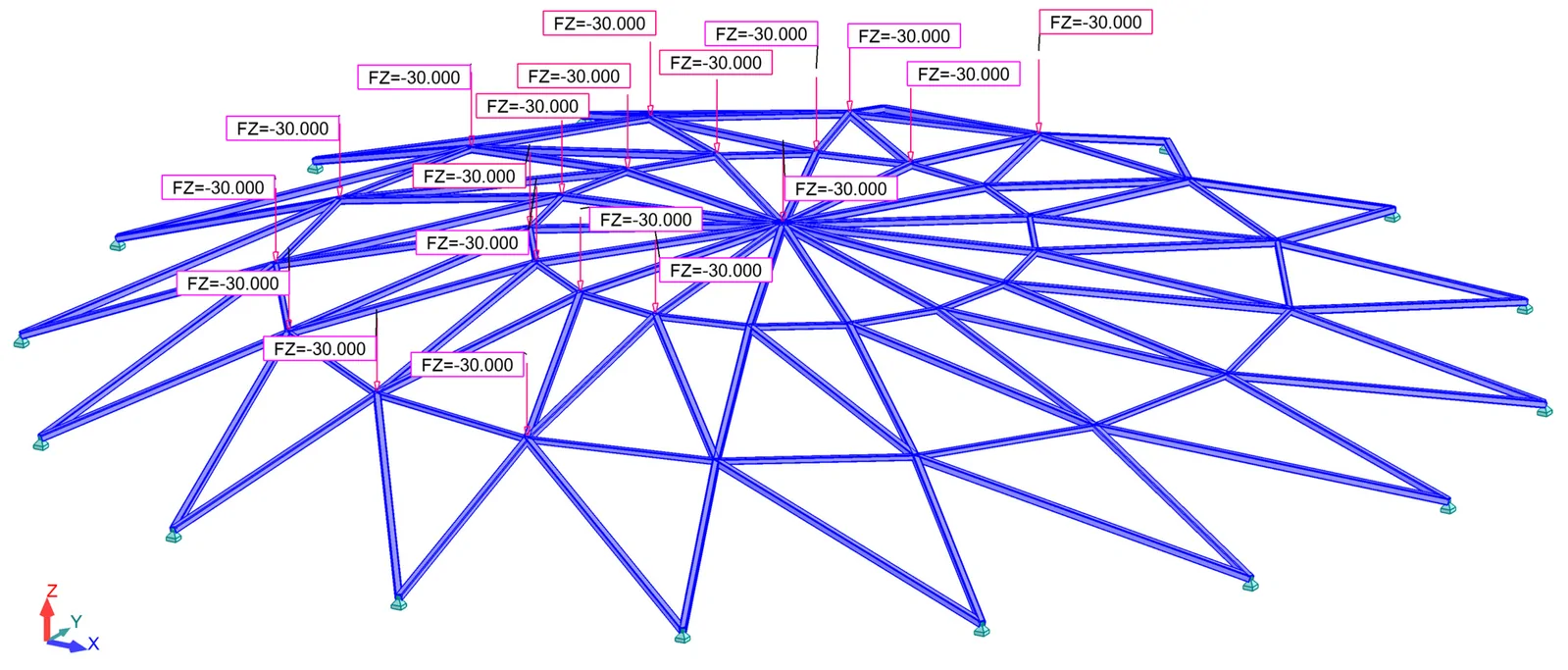
Structure asymmetric load diagram [kN].

Load Case 2—Asymmetric loading condition:

An asymmetric nodal loading was applied to a selected part of the dome surface (Figure 4), for example, to one half or a defined sector of the structure. This load case represents non-uniform snow accumulation, localized operational loading, or asymmetric environmental actions. Such loading conditions introduce additional transverse forces and bending effects, leading to increased variations in stress levels between different regions of the dome.

#### 2.2.5. Variants of Member Connections

Three variants of member connections at nodes were used in this study. In the first variant, corresponding to the pinned connection of the parallels and the bracing elements, bending moment releases were introduced at both ends of these elements. This means that at the beginning and end of the member, the rotations corresponding to the transfer of bending moments about the local axes of the element, i.e., My and Mz, were released. The elements retained the capacity to transfer axial forces and, depending on the element type, transverse forces, but did not transfer bending moments at the nodes. In the second variant, bending moment releases were applied only to the bracing elements. The parallels were left as continuous/frame elements capable of transferring bending moments. In practice, this means that for the members, the moments My and Mz were released at both ends of the element, while for the parallel elements, no end releases were introduced. In the third variant, all connections were modeled as rigid. No end releases were used for the parallel and bracing elements, which allowed the members to transfer both axial and transverse forces as well as bending moments at the nodes.

## 3. Results and Discussion

### 3.1. Asymmetrical Loading

#### 3.1.1. K1NS—Released Bracing and Ring Members

The structure was modeled as a spatial frame, taking into account the slack in the bracing and parallel bars. The application of these releases allowed the pinned behavior of the connections in these members to be simulated. Additionally, each dome member was subdivided into 10 finite elements in order to increase the accuracy of the FEM calculations. 

In the first stage, the structure was subjected to asymmetrical loading with nodal forces as shown in Figure 2.

##### Linear Static Analysis

The first step is to perform a linear static analysis. The integral forces and bearing capacities for the most stressed members are shown in Table 2. 

Based on the static and strength analysis, the following cross-sections were adopted for individual groups of bars (Table 3).

##### Buckling Analysis

**Figure 5:**
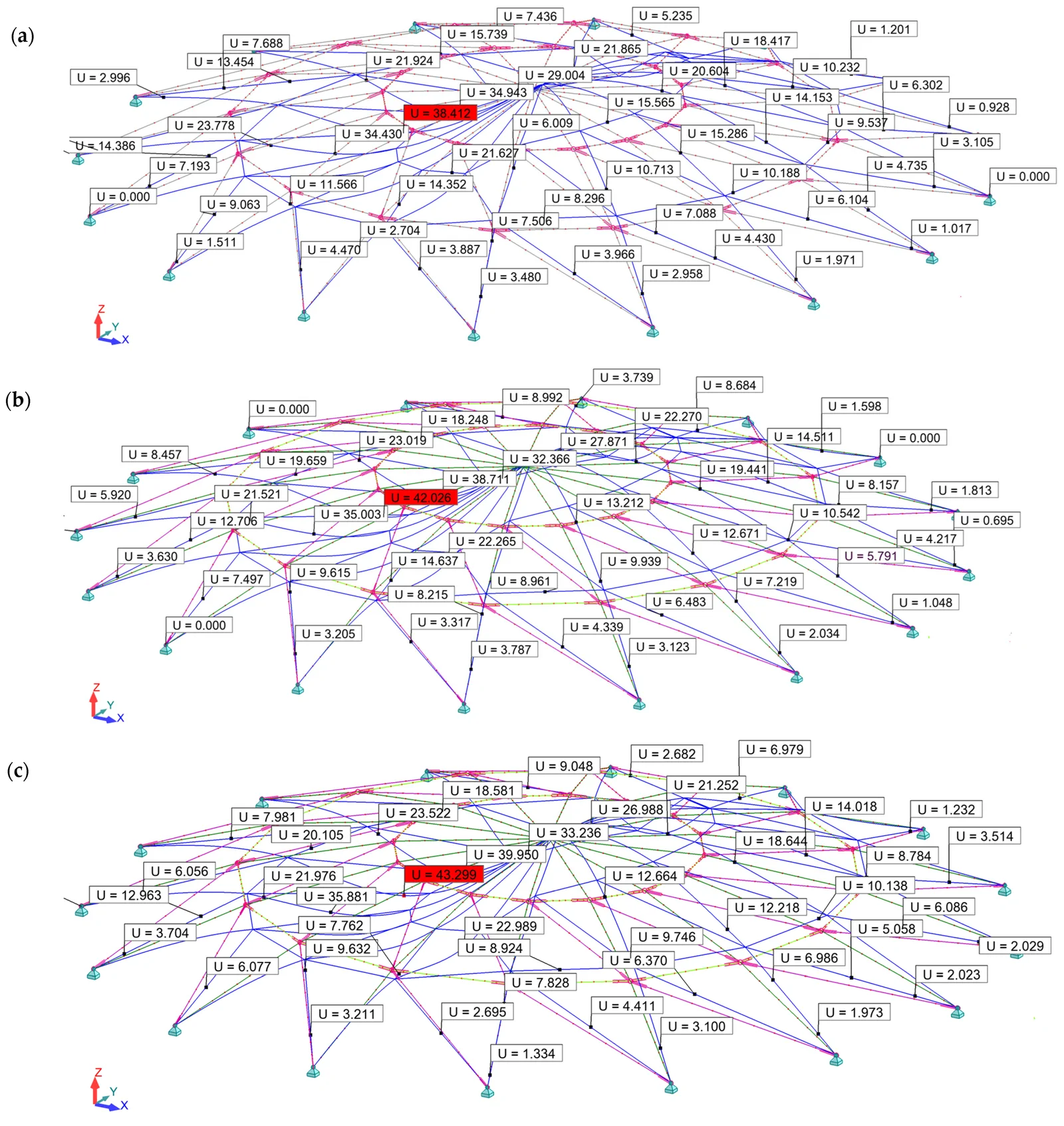
Structural deformation of the K1NS case: (**a**) LBA, (**b**) P-Δ, (**c**) GNA.

Based on the analysis, we can conclude that the cross-sections were correctly selected. In the next step, a linear buckling analysis was performed. The critical load multiplier was α^LBA^_cr_ = 1.48. According to standard provisions, a critical load multiplier less than 3.0 indicates the need to perform calculations incorporating a second-order analysis. Further analyses were conducted in two variants: a buckling analysis accounting for P-Δ effect and a GNA. The critical load multipliers for the individual cases were α^PΔ^_cr_ = 1.467 and α^GNA^_cr_ = 0.468, respectively. Taking the critical load multiplier from the LBA analysis as a reference value, the following relative errors were obtained: 0.875% for the P-Δ analysis and 68.394% for the GNA. Figure 5 shows the deformation of the structure for the individual analysis variants.

For all cases, the degree of utilization of the most critical members in each member group was verified (Table 4).

##### Modal Analysis

**Figure 6:**
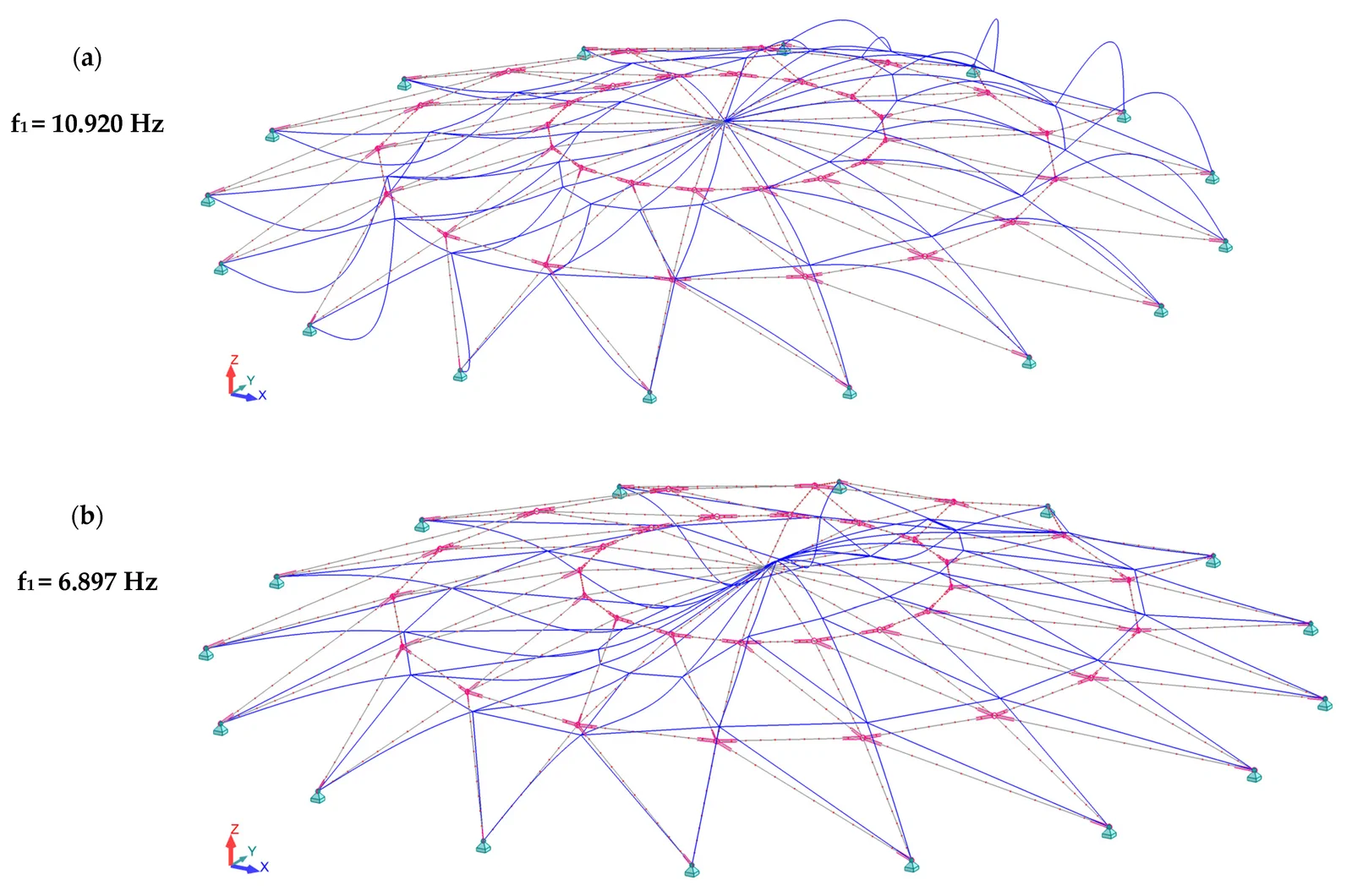
The first natural frequency and its form for the K1NS structure: (**a**) modal analysis and (**b**) modal analysis accounting for axial forces.

Additionally, a modal analysis and a modal analysis accounting for axial forces were performed for the presented structure. Table 5 and Table 6 summarize the first five frequencies for both cases, along with the percentage of current mass in each direction. The first natural frequency of the structure and its form for both cases are shown in Figure 6. 

Based on the obtained results, significant differences were observed between the dynamic response of the structure analyzed without and with axial forces. In the unloaded model, the first three vibration modes are characterized by a dominant mass fraction in the UX, UY, and UZ directions, respectively, indicating their global nature. Including axial forces from asymmetric loading redistributes the effective mass fractions among a significantly larger number of vibration modes and eliminates the dominance of the first modes. Analysis of the first 200 eigenmodes revealed that each mode exhibits a non-zero mass participation in all three translational directions, indicating the coupled spatial character of the vibrations and a significant modification of the modal structure. Furthermore, the cumulative modal mass reaches approximately 90% in the UZ direction only at the 178th eigenmode, whereas the corresponding cumulative mass participation in the UX and UY directions remains below 36%, demonstrating a much greater dispersion of the horizontal dynamic response.

To quantitatively assess the changes in the vibration mode shapes, the Modal Assurance Criterion (MAC) was determined, which quantifies the degree of similarity between the corresponding eigenmodes of the analyzed models (Table 7).

The obtained MAC values do not exceed 0.11, indicating very little similarity between the compared vibration modes. This confirms that taking into account axial forces from asymmetric loading leads not only to a change in the natural frequencies but also to a fundamental change in the vibration mode shapes and modal structure of the analyzed dome.

#### 3.1.2. K2NS—Released Bracing Members Only 

##### Linear Static Analysis

In the second case, a structure with analogous geometry and loading was considered. In this case, the structure was odelled as a spatial frame with pinned bracing elements. Again, the stresses in individual members were verified based on a linear static analysis (Table 8).

##### Buckling Analysis

**Figure 7:**
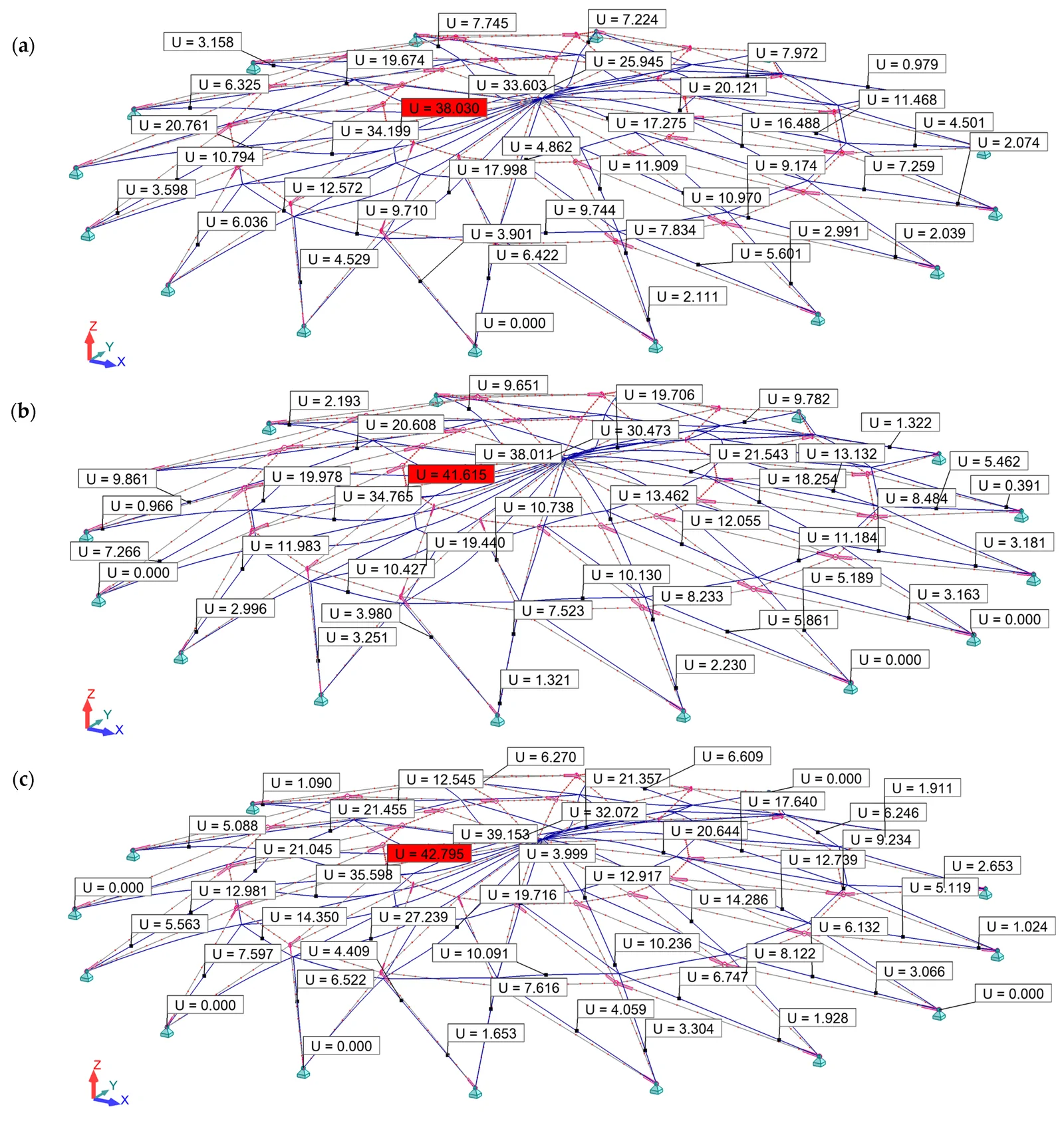
Structural deformation of the K2NS case: (**a**) LBA, (**b**) P-Δ, (**c**) GNA.

The change in the layout did not significantly affect the stresses in the individual structural members. In the second step, a linear buckling analysis was carried out. The critical load multiplier was α_cr_ = 1.503. The critical load multipliers for the P-Δ buckling analysis were α^PΔ^_cr_ = 1.49, whilst for the large-displacement buckling analysis they were α^GNA^_cr_ = 0.496. Taking the critical load multiplier from the LBA analysis as a reference value, the following relative errors were obtained: 0.880% for the P-Δ analysis and 67.014% for the GNA. Figure 7 shows the deformation of the structure for the individual analysis variants.

##### Modal Analysis

For this case, a modal analysis and a modal analysis taking axial forces into account were carried out again. Table 9 and Table 10 summarize the first five frequencies for both cases, along with the percentage of current mass in each direction. 

**Figure 8:**
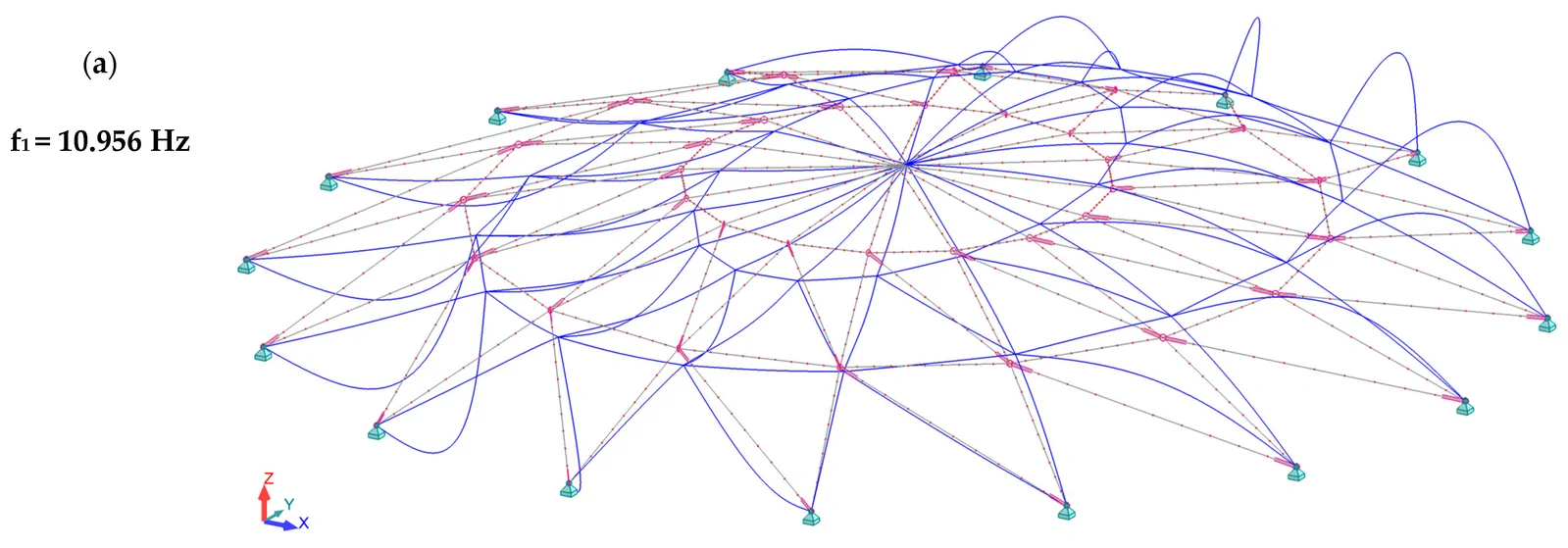

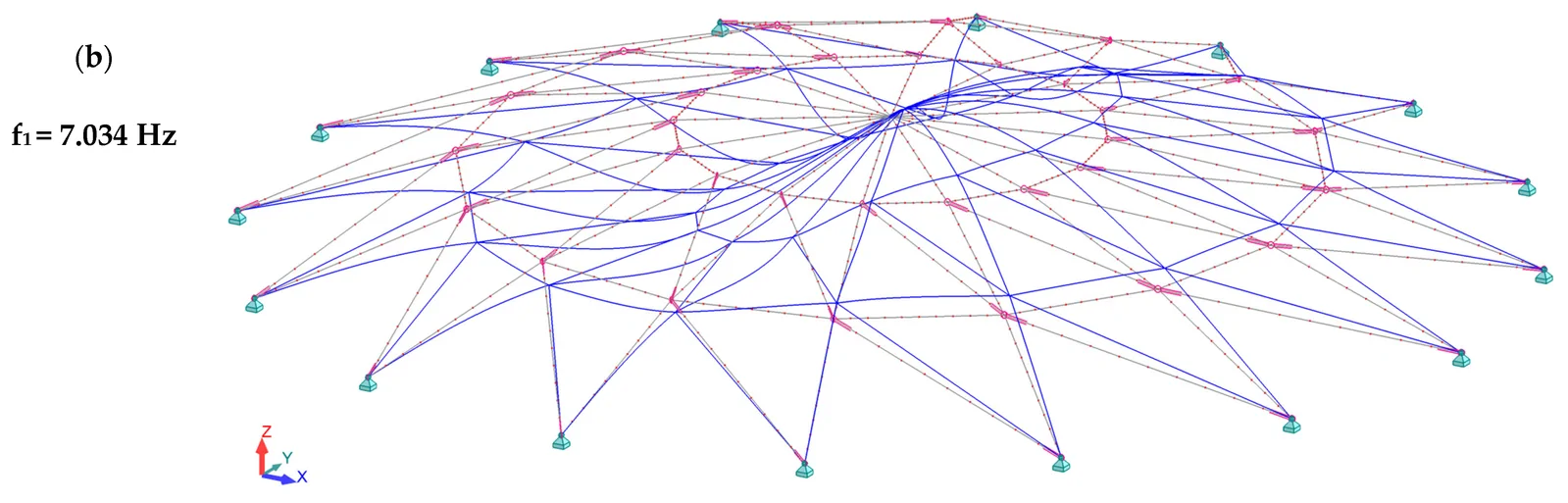
The first natural frequency and its form for the K2NS structure: (**a**) modal analysis and (**b**) modal analysis accounting for axial forces.

The first natural frequency of the structure and its form for both cases are shown in Figure 8.

Table 11 summarizes the values of the modal compliance coefficient MAC. 

Analysis of the results for the second case revealed trends similar to those for the first. Including axial forces resulted in a redistribution of effective mass fractions among a larger number of vibration modes, a loss of dominance of the first modes, and the appearance of coupled vibration mode shapes. Low MAC coefficient values (below 0.15) indicate a weak correlation between the corresponding vibration mode shapes of the models analyzed without and with axial forces. This confirms that changing the stress state affects not only the natural frequency values but also the vibration mode shapes.

#### 3.1.3. RNS—Fully Rigid Frame Model

##### Linear Static Analysis

In the final case, for asymmetric loading, a frame structure (with all connections treated as rigid) with the same geometry and loading was considered. The stresses in the individual members were verified on the basis of a linear static analysis (Table 12). 

##### Buckling Analysis

**Figure 9:**
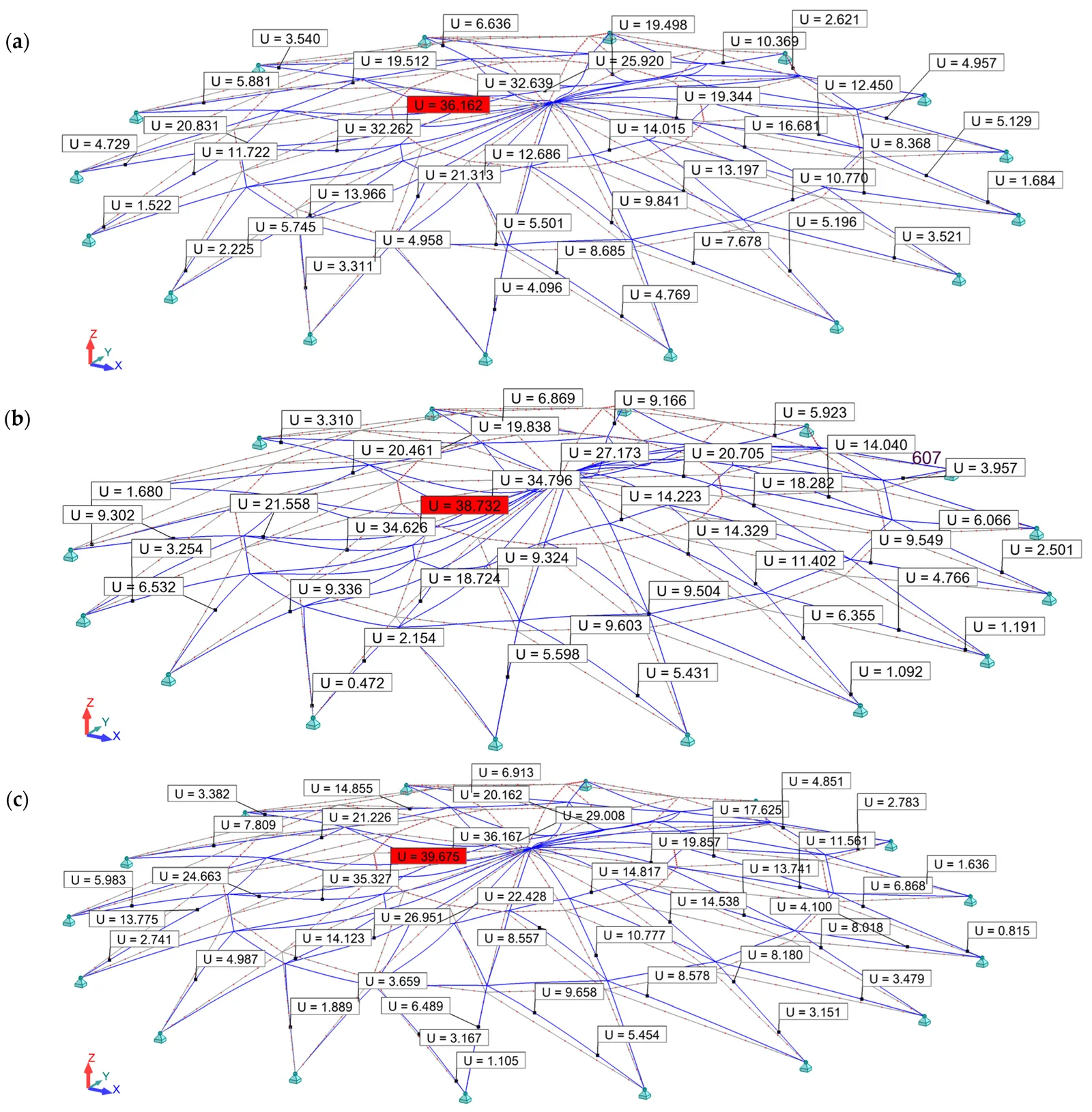
Structural deformation of the RNS case: (**a**) LBA, (**b**) P-Δ, (**c**) GNA.

Based on the linear buckling analysis, the critical load multiplier was determined to be α_cr_ = 3.261. In accordance with the provisions of the standards, for critical load multipliers in the range 3.0 ≤$\;\alpha_{cr}$ ≤ 10, it is recommended that the structure be verified using nonlinear analysis. In subsequent steps, a buckling analysis was performed taking into account P-Δ effect, and GNA. The critical load multipliers for the individual cases were α^PΔ^_cr_ = 3.187 and α^GNA^_cr_ = 2.150. Taking the critical load multiplier from the LBA analysis as a reference value, the following relative errors were obtained: 2.279% for the P-Δ analysis and 34.090% for the GNA. Figure 9 shows the deformation of the structure for the individual analysis variants.

##### Modal Analysis

In the following section, a modal analysis and a modal analysis taking axial forces into account were carried out. Table 13 and Table 14 summarize the first five frequencies for both cases, along with the percentage of current mass in each direction. 

**Figure 10:**
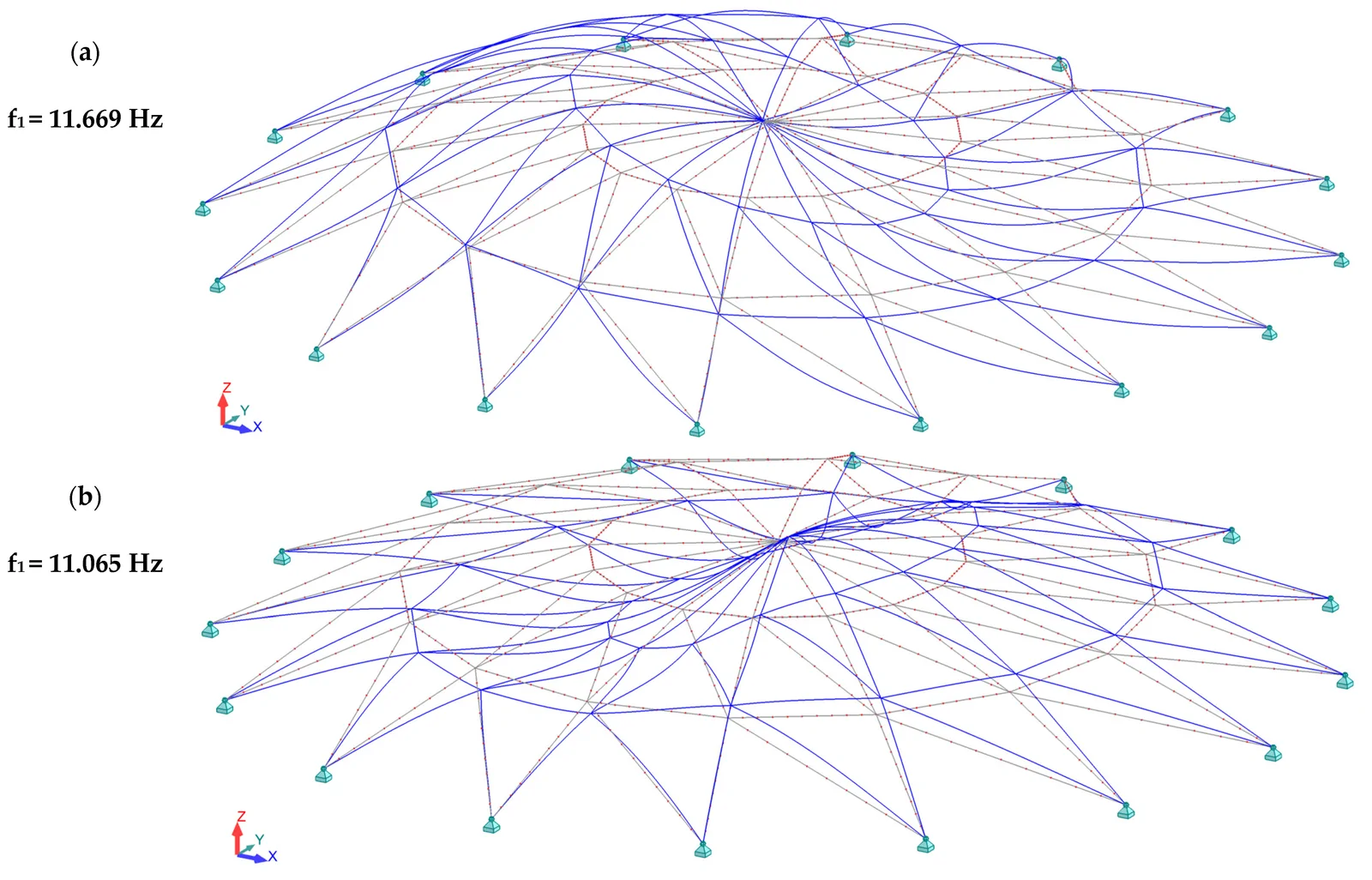
The first natural frequency and its form for the RNS structure: (**a**) modal analysis and (**b**) modal analysis accounting for axial forces.

The first natural frequency of the structure and its form for both cases are shown in Figure 10.

In the analyzed variant, a structural model with rigid connections between members was used. Compared with previous designs, considering axial forces does not significantly change the nature of the modal response. This demonstrates the preservation of the global nature of the basic vibration modes despite changes in the structure’s stress state. The natural frequency values change, but their decrease is relatively small and does not lead to a fundamental change in the nature of the dominant modes. The first two vibration modes, corresponding to horizontal displacements, retain their dominant mass fraction in the UX and UY directions, while the third mode remains primarily associated with the vertical UZ direction. After considering axial forces, only a partial redistribution of mass fractions between translation directions is observed, without losing the dominant nature of the basic modes. 

Table 15 summarizes the values of the modal compliance coefficient MAC. 

In contrast to the first case, moderate to high MAC values were obtained, reaching a maximum of 0.732. This indicates that the corresponding vibration mode shapes retain a significant degree of similarity despite the inclusion of axial forces. At the same time, the highest values of the coefficient occur not only on the matrix diagonal but also between the first and second vibration mode shapes, indicating a change in their order while maintaining a similar modal character. These results indicate that the influence of axial forces in the analyzed case leads primarily to a slight modification of the structure’s dynamic properties, without a fundamental reconstruction of its modal structure.

### 3.2. Symmetrical Load

#### 3.2.1. K1S—Released Bracing and Ring Members 

In the second stage, a structure subjected to symmetrical nodal forces at each node was considered, as shown in Figure 3. In this variant, the same cross-sections were used as for the non-uniform load.

##### Linear Static Analysis

In the first step, the section forces in the members and the stresses in the individual elements were verified using linear static analysis. Table 16 summarises the section forces and stresses for the most heavily stressed elements in each group of members.

##### Buckling Analysis

**Figure 11:**
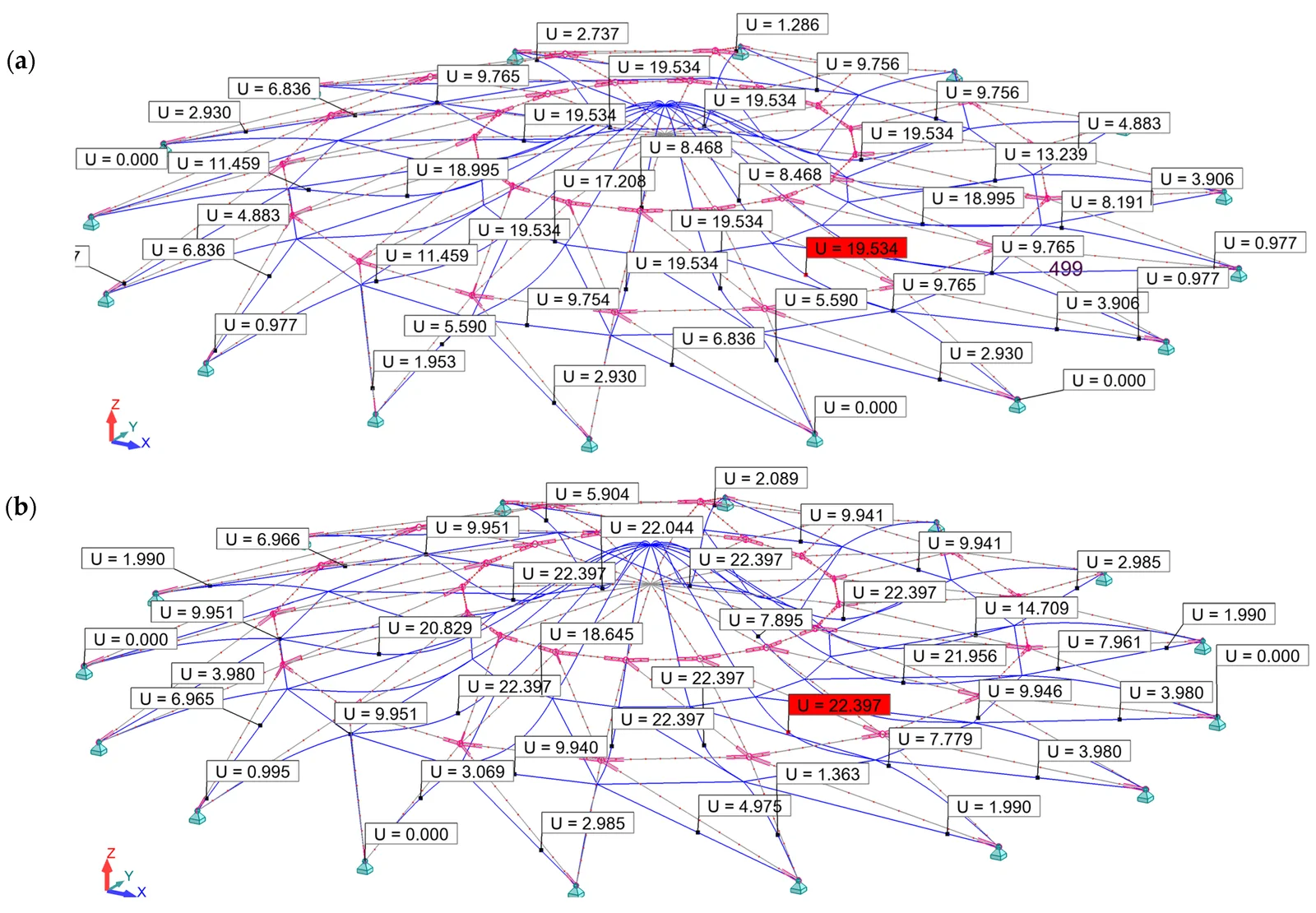

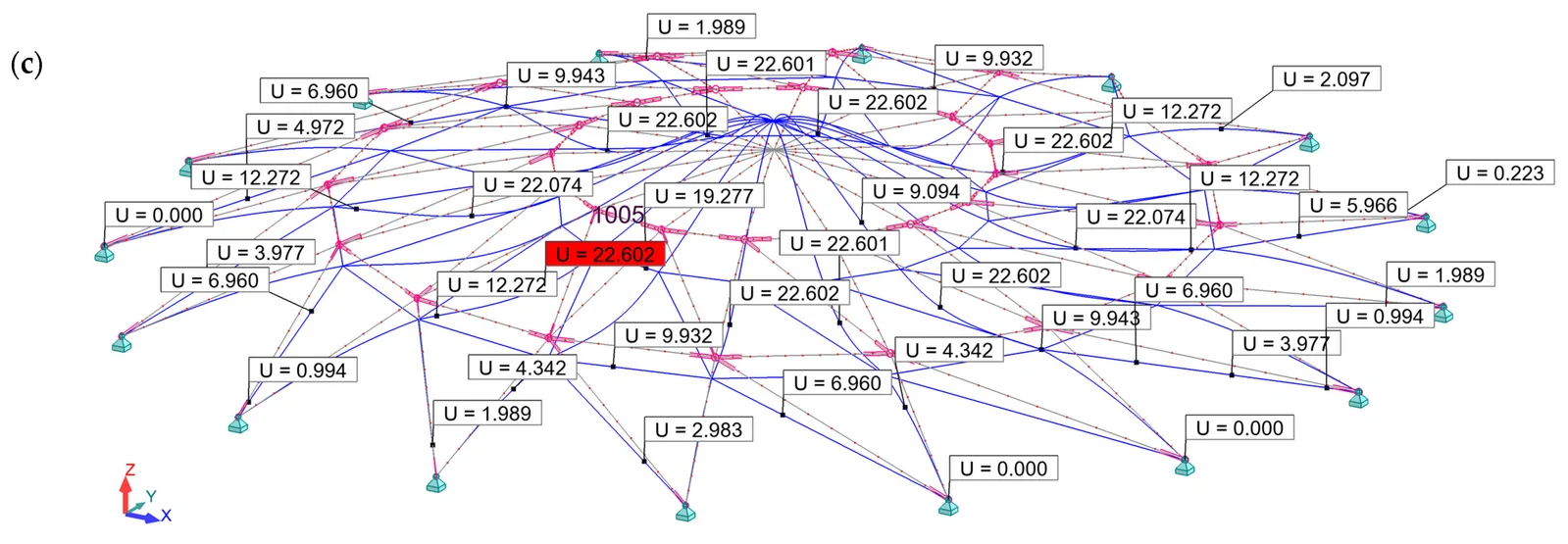
Structural deformation of the K1S case: (**a**) LBA, (**b**) P-Δ, (**c**) GNA.

In the next step, a linear buckling analysis was carried out. The critical load multiplier was found to be α^LBA^_cr_ = 2.698. As the critical load multiplier is less than 3.0, further analyses were carried out in two variants: a buckling analysis accounting for P-delta effect and a buckling analysis accounting for large displacements. The critical load multipliers for the individual cases were α^PΔ^_cr_ = 2.564 and α^GNA^_cr_ = 1.166, respectively. Taking the critical load multiplier from the LBA analysis as a reference value, the following relative errors were obtained: 4.963% for the P-Δ analysis and 56.782% for the GNA. Figure 11 shows the deformation of the structure for the individual analysis variants.

The stresses for the most heavily loaded members in each group of members are listed in Table 17. 

##### Modal Analysis

In the subsequent steps, a modal analysis and a modal analysis taking axial forces into account were carried out. Table 18 and Table 19 summarize the first five frequencies for both cases, along with the percentage of current mass in each direction.

**Figure 12:**
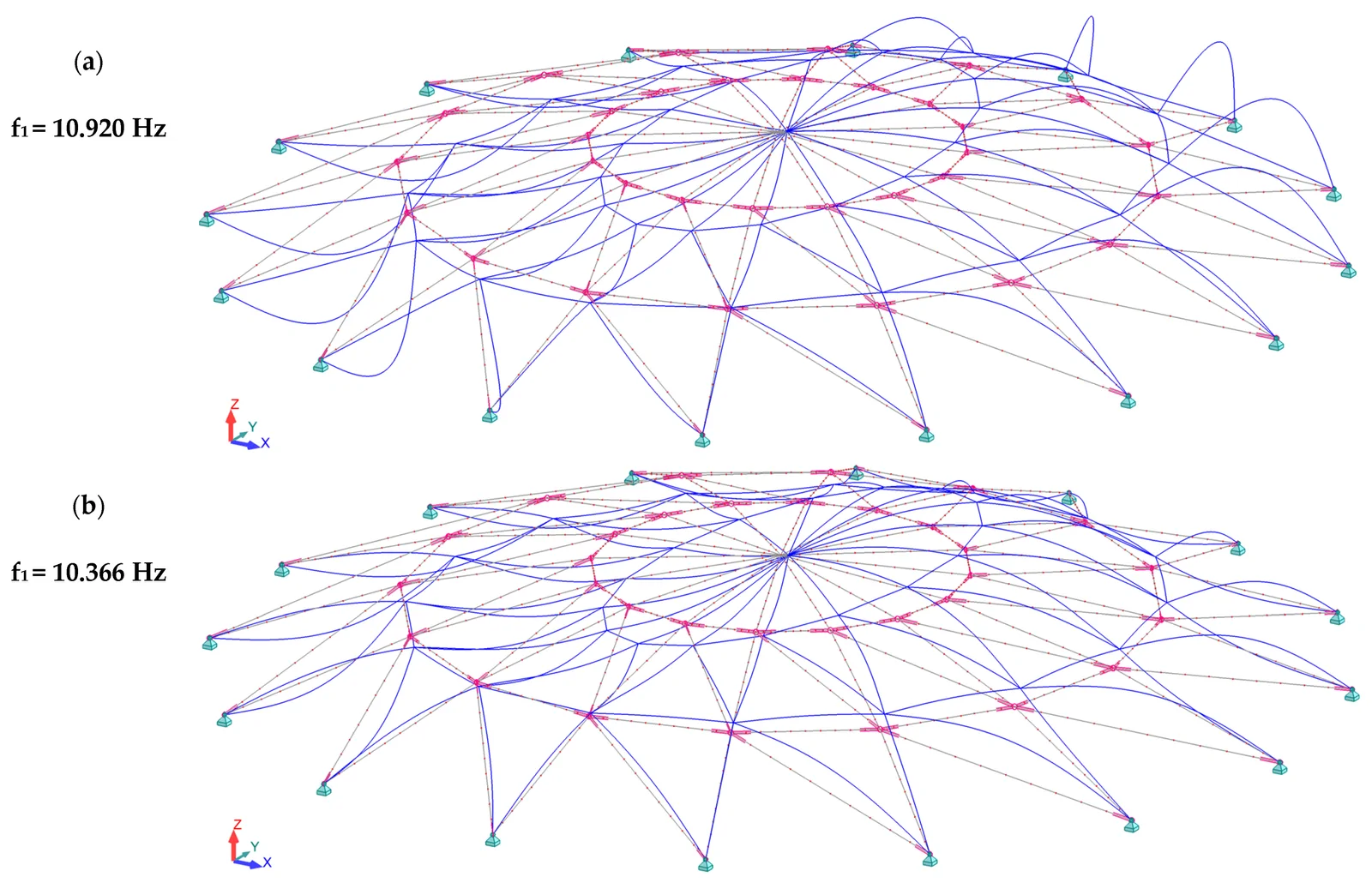
The first natural frequency and its form for the K1S structure: (**a**) modal analysis and (**b**) modal analysis accounting for axial forces.

Figure 12 shows the first natural frequency of the structure and its mode shape.

In the analyzed variant, the inclusion of axial forces resulted in only minor changes to the structure’s dynamic properties. A slight decrease in the natural frequency was observed, while their order and the dominant nature of the first three vibration modes were maintained. The first two modes are still primarily responsible for horizontal displacements in the UX and UY directions, while the third mode retains the dominant mass contribution in the vertical UZ direction. Table 20 summarizes the values of the modal compliance coefficient MAC. 

The obtained values indicate a high degree of similarity between the first three vibration modes before and after accounting for axial forces. For the first and second modes, the MAC coefficient is 0.743 and 0.743, respectively, while for the third mode, it reaches 0.976, indicating a virtually unchanged shape of this vibration mode. At the same time, the off-diagonal values remain low, confirming the lack of significant mixing of individual modes and their mutual independence. The obtained results indicate that the symmetric stress state induced by loading does not lead to a significant reconstruction of the structure’s modal structure, but only to a minor modification of the natural frequencies and mass fractions.

#### 3.2.2. K2S—Released Bracing Members Only

##### Linear Static Analysis

In the next case, a structure with identical geometry and loading was considered. The structure was modeled as a spatial frame pinned on the bracing elements. In the first step, the stresses in the individual members were verified using linear static analysis (Table 21).

##### Buckling Analysis

**Figure 13:**
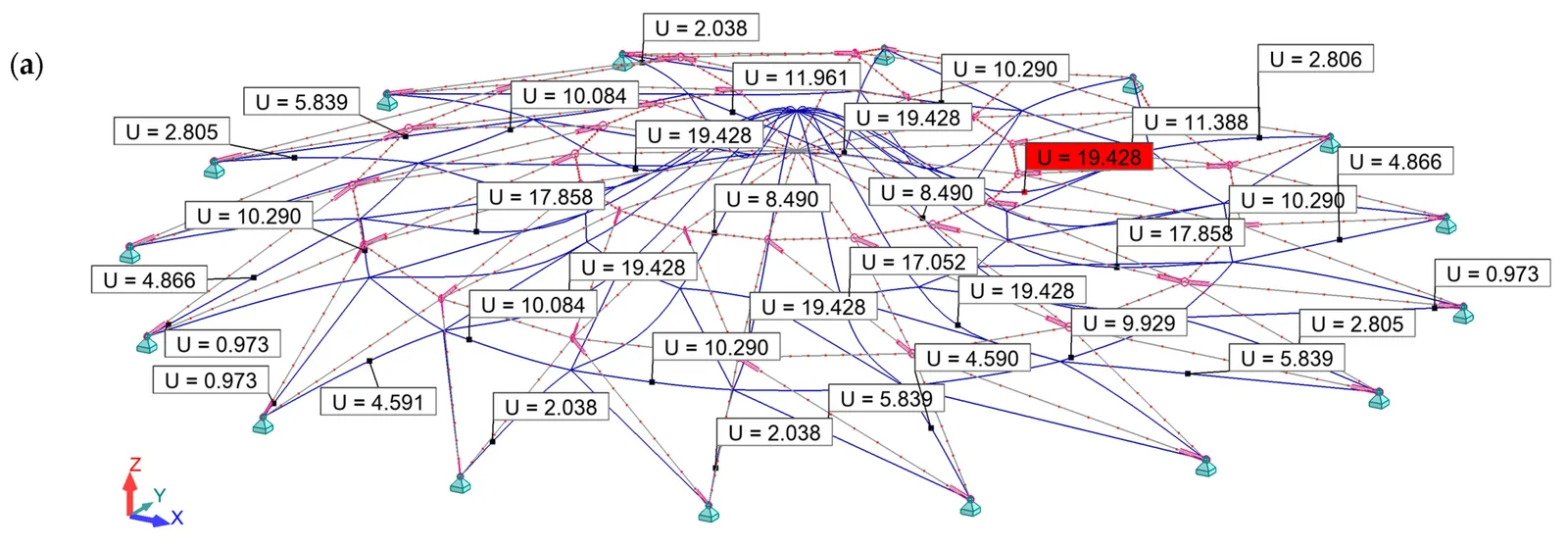

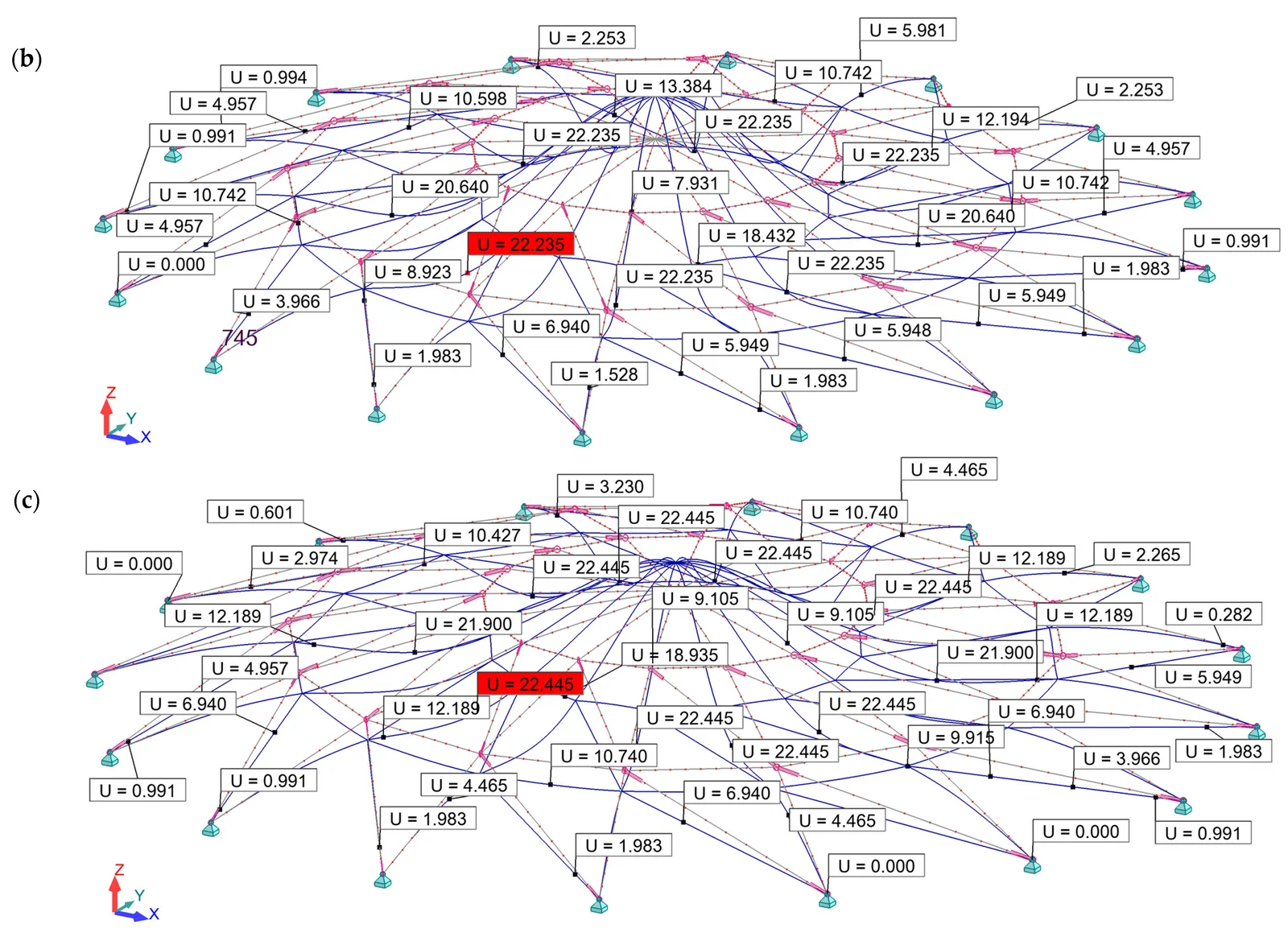
Structural deformation of the K2S case: (**a**) LBA, (**b**) P-Δ, (**c**) GNA.

A linear buckling analysis was then carried out, from which the critical load multiplier was determined to be α_cr_ = 2.766. Subsequent analyses again included: a buckling analysis accounting for P-Δ effect and GNA. The critical load multipliers for the individual cases were α^PΔ^_cr_ = 2.747 and α^GNA^_cr_ = 1.759. Taking the critical load multiplier from the LBA analysis as a reference value, the following relative errors were obtained: 0.696% for the P-Δ analysis and 36.427% for the GNA. Figure 13 shows the deformation of the structure for the individual analysis variants.

##### Modal Analysis

In the subsequent steps, a modal analysis and a modal analysis taking axial forces into account were carried out. Table 22 and Table 23 summarize the first five frequencies for both cases, along with the percentage of current mass in each direction.

**Figure 14:**
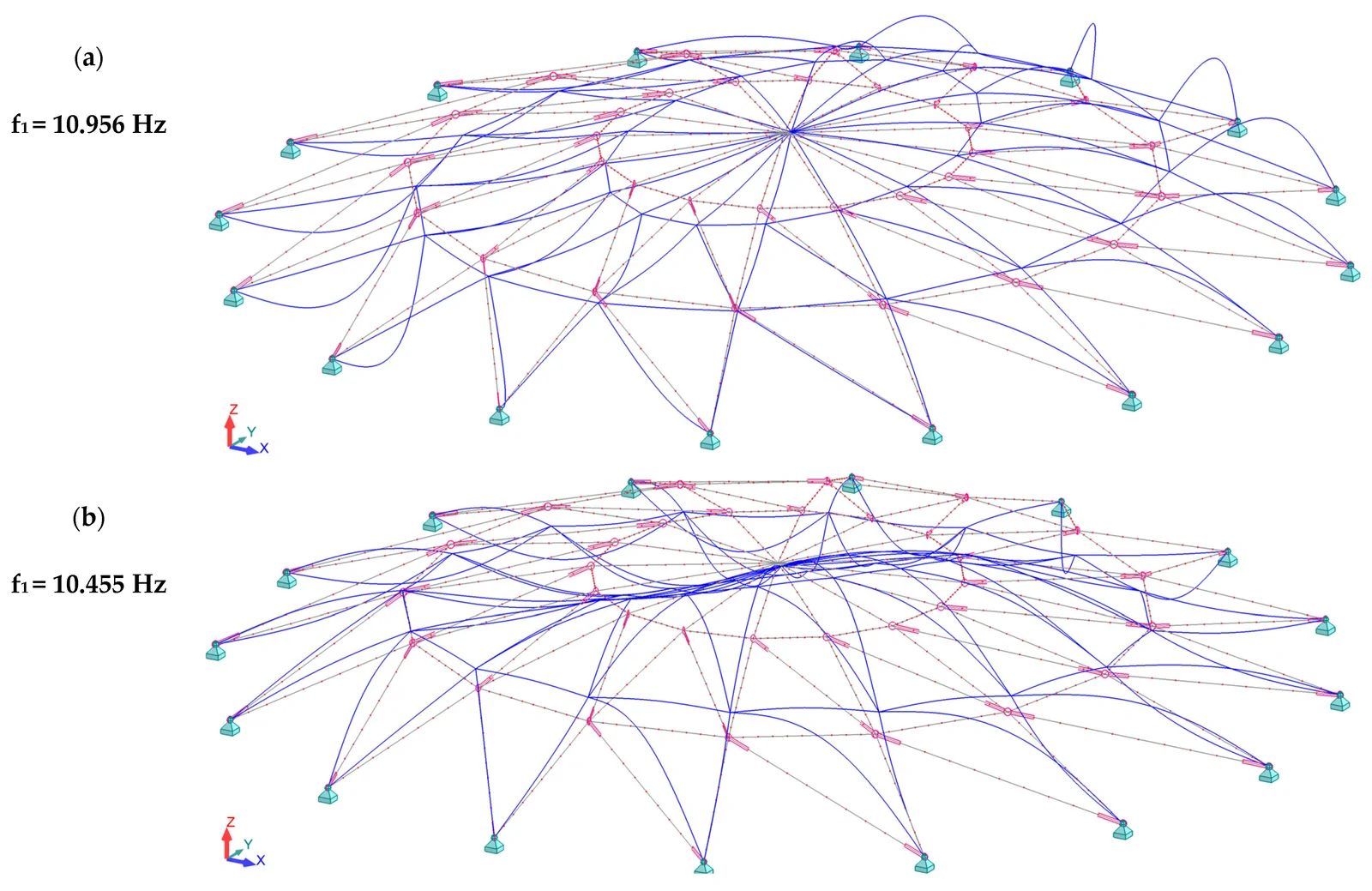
The first natural frequency and its form for the K2S structure: (**a**) modal analysis and (**b**) modal analysis accounting for axial forces.

The natural frequencies of the structure and the corresponding mode shapes were determined (Figure 14).

Table 24 summarizes the values of the modal compliance coefficient MAC. 

In the analyzed variant, considering axial forces induced by symmetrical loading resulted in only minor changes to the dynamic properties of the structure. A slight decrease in natural frequencies was observed, while the global nature of the basic vibration mode shapes was maintained. In the model without axial forces, the first two modes account for horizontal displacements in the UX and UY directions, with similar mass fractions. After considering axial forces, a slight redistribution of the effective mass fractions between the first two modes occurs, but their dominant nature is retained. The third vibration mode still accounts primarily for displacements in the vertical UZ direction, and its mass fraction decreases only slightly, indicating a minor effect of the stress state on the global dynamic properties of the structure.

#### 3.2.3. RS—Fully Rigid Frame Model

##### Linear Static Analysis

The final variant considered a frame structure (with all connections treated as rigid) with the same geometry and loading. The stresses in the individual members of the structure were verified on the basis of a linear static analysis (Table 25).

##### Buckling Analysis

**Figure 15:**
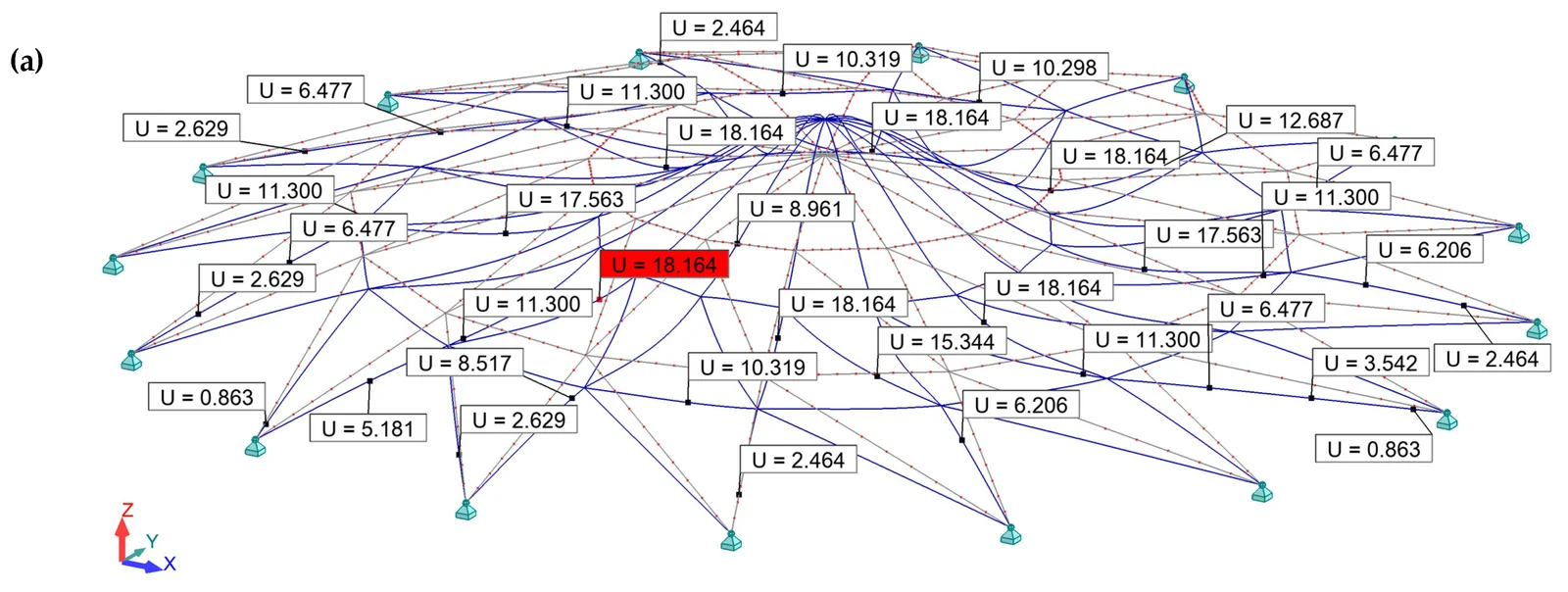

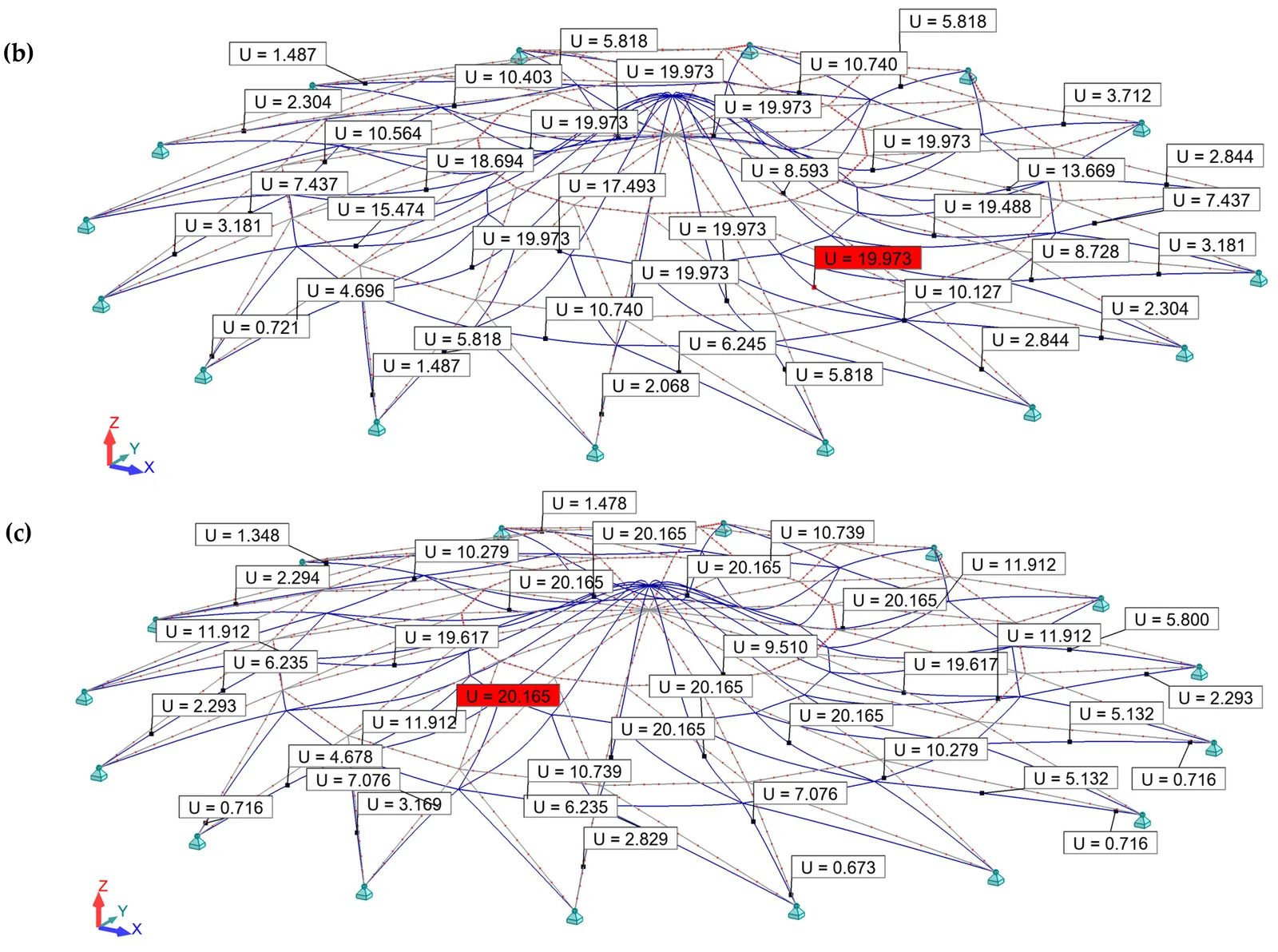
Structural deformation of the RS case: (**a**) LBA, (**b**) P-Δ, (**c**) GNA.

In the second step, a linear buckling analysis was carried out. The critical load multiplier was found to be α_cr_ = 3.730. In subsequent steps, a buckling analysis was performed taking into account both P-Δ effect and GNA. The critical load multiplier for the buckling analysis accounting for P-Δ effect was α^PΔ^_cr_ = 3.708. The critical load multiplier for the GNA was α^GNA^_cr_ = 2.703. Taking the critical load multiplier from the LBA analysis as a reference value, the following relative errors were obtained: 0.609% for the P-Δ analysis and 27.538% for the GNA. Figure 15 shows the deformation of the structure for the individual analysis variants.

##### Modal Analysis

For this case, a modal analysis and a modal analysis taking axial forces into account were carried out. Table 26 and Table 27 summarize the first five frequencies for both cases, along with the percentage of current mass in each direction. 

**Figure 16:**
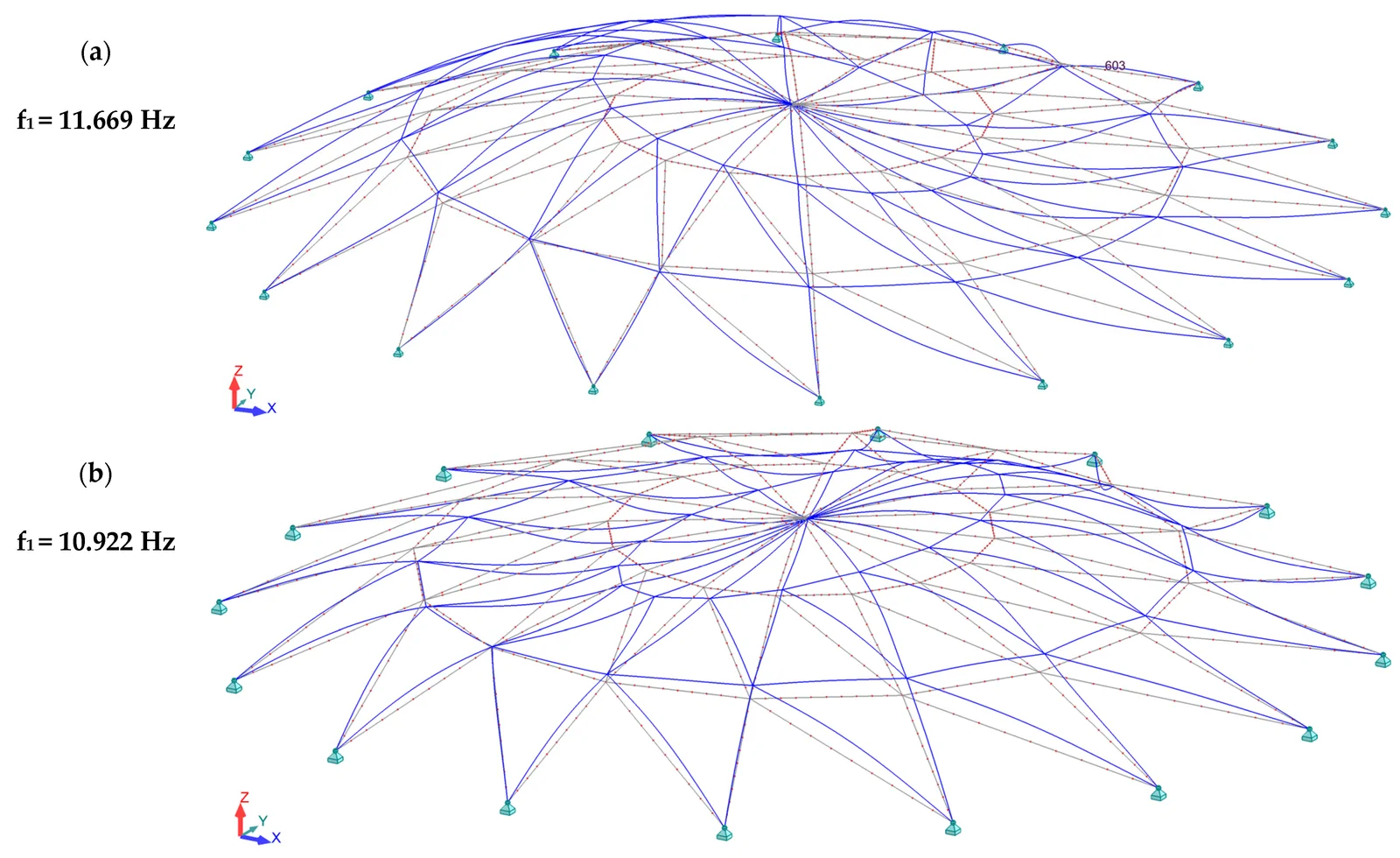
The first natural frequency and its form for the RS structure: (**a**) modal analysis and (**b**) modal analysis accounting for axial forces.

The first natural frequency of the structure and its form for both cases are shown in Figure 16.

In the analyzed variant, the inclusion of axial forces resulted in a slight reduction in the natural frequencies, while maintaining the global nature of the basic vibration modes. The first two modes still account for displacements in the UX and UY directions, respectively, maintaining an almost completely independent nature. Unlike the cases analyzed for asymmetric loading, no significant coupling of translation directions or dispersion of effective mass fractions among a larger number of vibration modes is observed. Table 28 summarizes the values of the modal compliance coefficient MAC. 

Analysis of the MAC modal consistency coefficient confirms the very high similarity of the corresponding vibration mode shapes. The coefficient values on the matrix diagonal range from 0.93 to 0.97, while the off-diagonal values remain close to zero. This indicates that including axial forces did not significantly change the vibration mode shapes or their relative order. The obtained results indicate that the symmetric stress state primarily affects the slight change in natural frequencies and the redistribution of mass fractions, while maintaining the structure’s modal structure virtually unchanged.

## 4. Conclusions

The conducted studies have shown that the static and dynamic response of a low-rise steel dome is largely determined by both the loading pattern and the method of modeling the nodal connections, as well as the level of sophistication of the numerical analysis. The structure’s greatest sensitivity was observed for asymmetric loading. In the model with pinned parallel and truss connections, incorporating a full geometrically nonlinear analysis reduced the critical load multipliers from 1.48 to 0.468, corresponding to a 68.4% decrease. This means that actual stability loss occurs at approximately 46.8% of the load corresponding to linear buckling analysis. This result clearly indicates that classical linear analysis significantly overestimates the structure’s load-bearing capacity under asymmetric loads. The use of rigid nodal connections significantly increases the dome’s resistance to stability loss. For the frame model, the critical coefficient decreased from 3.261 to 2.150, which corresponds to a significantly smaller decrease than in the pinned models. This confirms the significant influence of connection stiffness on the global stability of low-rise bar domes. The most original result of this work is from the modal analysis that takes into account the influence of axial forces. For asymmetric loading, incorporating the geometric stiffness matrix leads not only to a reduction in the first natural frequency (from 10.956 Hz to 7.034 Hz) but, above all, to a fundamental change in the modal structure of the structure. In the model without axial forces, the first three vibration modes are global in nature and correspond to dominant displacements in the X, Y, and Z directions. After accounting for axial forces, the dynamic energy is dispersed among a large number of vibration modes, and the dominance of the first modes disappears. Analysis of the first 200 modes showed that each mode has a non-zero mass contribution in all translational directions, indicating strong spatial coupling of the dynamic response. The fundamental change in the vibration character is further confirmed by the very low values of the Modal Assurance Criterion (MAC < 0.11). This means that after considering axial forces, there is not only a change in the mode order or a reduction in the natural frequencies, but also the creation of qualitatively new vibration modes. Classical bending and torsional modes are replaced by complex bending–torsional modes resulting from the asymmetric stress distribution and local stiffness loss of the compressed elements. Consequently, modal analysis with a geometric matrix describes a different dynamic state of the structure than classical modal analysis. A different mechanism of action is observed for symmetrical loading. The decrease in critical load multipliers is significantly smaller than for asymmetric loading, and the effect of axial forces on dynamic properties is limited primarily to a reduction in the natural frequencies. In models with rigid connections, MAC values reach approximately 0.986, confirming the preservation of virtually identical vibration modes. This means that the stress state reduces the effective stiffness of the structure without significantly altering the deformation mechanism. Only in models with pinned connections is a partial loss of compliance of the vibration mode shapes observed. The obtained results show that asymmetric loading is a factor determining both the stability safety and the dynamic response of low-rise bar domes. Under such conditions, the use of classical modal analysis and linear buckling analysis can lead to incorrect assessment of the structure’s behavior. A reliable analysis requires accounting for the influence of axial forces via the geometric stiffness matrix and the use of a full geometrically nonlinear analysis. The presented results also indicate that modal analysis taking into account the geometric stiffness matrix can be a valuable diagnostic tool for identifying loss of spatial stiffness of the structure. The observed change in modal structure and the dispersion of mass fractions can be considered indicators of the structure approaching a state of stability loss, which opens new possibilities for the use of modal analysis in assessing the safety of spatial bar structures.

## 5. Practical Design Recommendations

Based on the obtained results, the following practical recommendations can be formulated for the analysis and design of low-rise steel domes:

1. Linear buckling analysis (LBA) should not be used as the sole basis for stability assessment under asymmetric loading. For the dome with pinned joints, the critical load multiplier obtained from LBA overestimated the actual load-carrying capacity by more than twofold. Therefore, LBA should be regarded only as a preliminary assessment tool.
2. Full geometrically nonlinear analysis (GNA) is recommended whenever significant asymmetric loading may occur. Such loading produces highly non-uniform axial force distributions that substantially reduce the structural stiffness and critical load.
3. The influence of geometric stiffness should be included in modal analyses whenever the structure is subjected to considerable compressive forces. Neglecting axial forces may lead to incorrect prediction of both the natural frequencies and the vibration mode shapes.
4. The Modal Assurance Criterion (MAC) should be evaluated when comparing modal properties of prestressed and unstressed structures. Low MAC values indicate that the vibration mechanism has fundamentally changed and that direct comparison of corresponding modes is no longer valid.
5. Pinned joint configurations require particular attention during design. They exhibited the largest reduction in both critical load multiplier and dynamic stiffness, making them considerably more sensitive to asymmetric loading than rigidly connected domes.
6. Rigid joints improve the robustness of the structural system. Their higher rotational stiffness limits the redistribution of internal forces and reduces the sensitivity of the dome to geometric nonlinearity.
7. Asymmetric load cases should be explicitly considered during design verification. Snow drift, partial maintenance loads, temporary construction stages, or local equipment loads may generate stress distributions similar to those investigated in this study.
8. The significant redistribution of modal mass observed under asymmetric loading suggests that dynamic analyses should not be limited to the first few vibration modes. A substantially larger number of modes may be required to correctly represent the dynamic response of prestressed steel domes.
9. Design codes may underestimate the effects of asymmetric loading if analyses are limited to linear procedures. For slender steel domes, the combined consideration of second-order effects, geometric nonlinearity, and geometric stiffness provides a substantially more reliable assessment of structural safety.

## Figures and Tables

**Table 1:** Limiting chemical composition of S355J2H steel according to PN-EN 10210-1.

Steel [% Mass Content]						
Element	C	Si	Mn	P	S	N
Maximum content [%]	≤0.20	≤0.55	≤1.60	≤0.030	≤0.030	≤0.009

**Table 2:** Summary of internal forces for the most stressed members for the K1NS case.

Internal Force/ Bearing Capacity	Meridians Member No. 53	R1 Member No. 1	R2 Member No. 17	Bracing Member No. 92
N_Ed_ [kN]	283.19	301.59	145.50	142.69
M_y_ [kNm]	2.34	-	-	-
N_c,Rd_ [kN]	908.80	450.85	450.85	702.90
N_b,Rd_ [kN]	405.87	393.60	214.98	185.39
M_y,c,Rd_ [kNm]	26.19	-	-	-
U_H,max_ [mm]	8.35			
U_H,dop_ [mm]	13.33			
U_V,max_ [mm]	36.54			
U_V,dop_ [mm]	66.67			

N_Ed_ [kN]—axial force, M_y_—moments M_y_, N_c,Rd_ [kN]—design cross-sectional resistance under uniform compression, N_b,Rd_ [kN]—design buckling resistance of the compression element, U_H,max_—maximum horizontal displacement [mm], U_H,dop_—allowable horizontal displacement [mm]—H/150, U_V,max_ [mm]—maximum vertical displacement [mm], U_V,dop_—allowable vertical displacement [mm]—D/300.

**Table 3:** Summary of the adopted cross-sections and utilization ratio for individual groups for the K1NS case.

Group	Profile	Utilization Ratio
Meridians	RK 90 × 90 × 8	84
Parallels—R1	RK 70 × 70 × 5	77
Parallels—R2	RK 70 × 70 × 5	68
Bracing	RK 90 × 90 × 6	77

**Table 4:** Summary of utilization ratios for individual member groups for the K1NS case.

Group	Utilization Ratios		
	Linear Statics	P-Δ	GNA
Meridians	84	94	94
Parallels—R1	77	83	83
Parallels—R2	68	67	68
Bracing	77	78	78

**Table 5:** Frequency and current masses for modal analysis for the K1NS case.

Mode	Frequency (Hz)	Current Mass [%]		
	Modal Analysis	UX	UY	UZ
1	10.920	6.083	1.404	0.000
2	10.921	1.405	6.084	0.000
3	11.537	0.000	0.000	17.647
4	11.852	0.000	0.000	0.000
5	11.854	0.000	0.000	0.000

**Table 6:** Frequency and current masses for modal analysis accounting for axial forces for the K1NS case.

Mode	Frequency (Hz)	Current Mass [%]		
	Modal Analysis Accounting for Axial Forces	UX	UY	UZ
1	6.897	0.014	0.173	1.009
2	6.954	0.355	0.611	0.044
3	8.227	0.002	0.276	1.074
4	8.336	0.742	0.244	0.043
5	9.451	0.090	0.502	1.248

**Table 7:** Comparison of MAC values for the first five mode shapes for the K1NS case.

		Modal Analysis Accounting for Axial Forces				
	MAC	Mode 1	Mode 2	Mode 3	Mode 4	Mode 5
**Modal analysis**	Mode 1	0.0001	0.0002	0.0114	0.0002	0.0001
	Mode 2	0.0525	0.0000	0.0580	0.0001	0.0525
	Mode 3	0.0557	0.0000	0.0588	0.0000	0.0557
	Mode 4	0.1023	0.0000	0.0124	0.0002	0.1023
	Mode 5	0.0214	0.0002	0.1070	0.0001	0.0214

**Table 8:** Summary of the adopted cross-sections and utilization ratio for individual groups for the K2NS case.

Group	Cross-Section	Utilization Ratio
Meridians	RK 90 × 90 × 8	85
Parallels—R1	RK 70 × 70 × 5	81
Parallels—R2	RK 70 × 70 × 5	75
Bracing	RK 90 × 90 × 6	76

**Table 9:** Frequency and current masses for modal analysis for the K2NS case.

Mode	Frequency (Hz)	Current Mass [%]		
	Modal Analysis	UX	UY	UZ
1	10.956	2.563	4.717	0.002
2	10.956	4.778	2.708	0.000
3	11.556	0.000	0.000	17.385
4	11.872	0.003	0.001	0.000
5	11.873	0.000	0.001	0.000

**Table 10:** Frequency and current masses for modal analysis accounting for axial forces for the K2NS case.

Mode	Frequency (Hz)	Current Mass [%]		
	Modal Analysis Accounting for Axial Forces	UX	UY	UZ
1	7.034	0.015	0.181	1.028
2	7.091	0.360	0.612	0.041
3	8.224	0.002	0.270	1.061
4	8.324	0.745	0.247	0.042
5	9.497	0.095	0.491	1.277

**Table 11:** Comparison of MAC values for the first five mode shapes for the K2NS case.

		Modal Analysis Accounting for Axial Forces				
	MAC	Mode 1	Mode 2	Mode 3	Mode 4	Mode 5
**Modal analysis**	Mode 1	0.0149	0.0003	0.0463	0.0000	0.1217
	Mode 2	0.0471	0.0003	0.0274	0.0007	0.0107
	Mode 3	0.0577	0.0000	0.0598	0.0000	0.0674
	Mode 4	0.0008	0.0001	0.0611	0.0003	0.0984
	Mode 5	0.1434	0.0020	0.0609	0.0005	0.0049

**Table 12:** Summary of the adopted cross-sections and utilization ratio for individual groups for the RNS case.

Group	Cross-Section	Utilization Ratio
Meridians	RK 90 × 90 × 8	76
Parallels—R1	RK 70 × 70 × 5	86
Parallels—R2	RK 70 × 70 × 5	76
Bracing	RK 90 × 90 × 6	84

**Table 13:** Frequency and current masses for modal analysis for the RNS case.

Mode	Frequency (Hz)	Current Mass [%]		
	Modal Analysis	UX	UY	UZ
1	11.669	7.267	0.113	0.000
2	11.669	0.114	7.278	0.000
3	13.057	0.000	0.000	7.815
4	14.190	0.018	2.921	0.000
5	14.191	2.946	0.014	0.000

**Table 14:** Frequency and current masses for modal analysis accounting for axial forces for the RNS case.

Mode	Frequency (Hz)	Current Mass [%]		
	Modal Analysis Accounting for Axial Forces	UX	UY	UZ
1	11.065	0.916	4.336	0.010
2	11.093	4.373	1.203	0.015
3	12.469	1.915	0.039	6.741
4	12.967	0.001	3.977	0.059
5	13.450	2.103	0.012	1.566

**Table 15:** Comparison of MAC values for the first five mode shapes for the RNS case.

		Modal Analysis Accounting for Axial Forces				
	MAC	Mode 1	Mode 2	Mode 3	Mode 4	Mode 5
**Modal analysis**	Mode 1	0.2323	0.6752	0.0787	0.0192	0.0005
	Mode 2	0.7173	0.2477	0.0077	0.0108	0.0027
	Mode 3	0.0014	0.0395	0.7200	0.0382	0.0933
	Mode 4	0.0515	0.0038	0.0349	0.7323	0.0115
	Mode 5	0.0126	0.0341	0.1133	0.0225	0.5556

**Table 16:** Summary of internal forces for the most stressed members for the K1S case.

Internal Force/ Bearing Capacity	Meridians Member No. 60	R1 Member No. 13	R2 Member No. 26	Bracing Member No. 100
N_Ed_ [kN]	157.203	184.387	74.741	0.308
M_y_ [kNm]	4.684			
N_c.Rd_ [kN]	908.80	450.850	450.850	702.900
N_b.Rd_ [kN]	405.868	393.596	214.977	266.546
M_y.c.Rd_ [kNm]	26.190	-	-	-
Utilization ratio [%]	61	47	35	0
U_H.max_ [mm]	3.56			
U_H.dop_ [mm]	13.33			
U_V.max_ [mm]	16.84			
U_V.dop_ [mm]	66.67			

**Table 17:** Summary of utilization ratios for individual member groups for the K1S case.

Group	Utilization Ratios		
	Linear Statics	P-Δ	GNA
Meridians	61	62	62
Parallels—R1	47	53	54
Parallels—R2	35	36	35
Bracing	0	0	0

**Table 18:** Frequency and current masses for modal analysis for the K1S case.

Mode	Frequency (Hz)	Current Mass [%]		
	Modal Analysis	UX	UY	UZ
1	10.920	3.786	3.669	0.000
2	10.921	3.650	3.868	0.000
3	11.537	0.000	0.000	17.626
4	11.852	0.000	0.000	0.000
5	11.854	0.001	0.005	0.000

**Table 19:** Frequency and current masses for modal analysis accounting for axial forces for the K1S case.

Mode	Frequency (Hz)	Current Mass [%]		
	Modal Analysis Accounting for Axial Forces	UX	UY	UZ
1	10.366	5.234	0.851	0.000
2	10.366	0.821	5.207	0.000
3	11.344	0.001	0.001	17.474
4	11.536	1.173	0.285	0.002
5	11.538	0.335	1.239	0.000

**Table 20:** Comparison of MAC values for the first five mode shapes for the K1S case.

		Modal Analysis Accounting for Axial Forces				
	MAC	Mode 1	Mode 2	Mode 3	Mode 4	Mode 5
**Modal analysis**	Mode 1	0.7433	0.1307	0.0000	0.0484	0.0029
	Mode 2	0.1311	0.7426	0.0000	0.0036	0.0500
	Mode 3	0.0000	0.0000	0.9755	0.0000	0.0001
	Mode 4	0.0000	0.0000	0.0001	0.0001	0.0001
	Mode 5	0.0000	0.0000	0.0000	0.0002	0.0001

**Table 21:** Summary of the adopted cross-sections and utilization ratio for individual groups for the K2S case.

Group	Cross-Section	Utilization Ratio
Meridians	RK 90 × 90 × 8	61
Parallels—R1	RK 70 × 70 × 5	52
Parallels—R2	RK 70 × 70 × 5	38
Bracing	RK 90 × 90 × 6	0

**Table 22:** Frequency and current masses for modal analysis for the K2S case.

Mode	Frequency (Hz)	Current Mass [%]		
	Modal Analysis	UX	UY	UZ
1	10.956	2.563	4.717	0.002
2	10.956	4.778	2.708	0.000
3	11.556	0.000	0.000	17.385
4	11.872	0.003	0.001	0.000
5	11.873	0.000	0.001	0.000

**Table 23:** Frequency and current masses for modal analysis accounting for axial forces for the K2S case.

Mode	Frequency (Hz)	Current Mass [%]		
	Modal Analysis Accounting for Axial Forces	UX	UY	UZ
1	10.455	3.264	2.973	0.000
2	10.455	2.926	3.291	0.000
3	11.378	0.000	0.000	17.165
4	11.640	0.499	0.716	0.000
5	11.641	0.636	0.528	0.001

**Table 24:** Comparison of MAC values for the first five mode shapes for the K2S case.

		Modal Analysis Accounting for Axial Forces				
	MAC	Mode 1	Mode 2	Mode 3	Mode 4	Mode 5
**Modal analysis**	Mode 1	0.0122	0.8751	0.0000	0.0011	0.0354
	Mode 2	0.8740	0.0122	0.0000	0.0356	0.0010
	Mode 3	0.0000	0.0001	0.9783	0.0001	0.0000
	Mode 4	0.0001	0.0001	0.0000	0.0002	0.0002
	Mode 5	0.0000	0.0000	0.0000	0.0001	0.0005

**Table 25:** Summary of the adopted cross-sections and utilization ratio for individual groups for the RS case.

Group	Cross-Section	Utilization Ratio
Meridians	RK 90 × 90 × 8	56
Parallels—R1	RK 70 × 70 × 5	53
Parallels—R2	RK 70 × 70 × 5	40
Bracing	RK 90 × 90 × 6	8

**Table 26:** Frequency and current masses for modal analysis for the RS case.

Mode	Frequency (Hz)	Current Mass [%]		
	Modal Analysis	UX	UY	UZ
1	11.669	7.267	0.113	0.000
2	11.669	0.114	7.278	0.000
3	13.057	0.000	0.000	7.815
4	14.190	0.018	2.921	0.000
5	14.191	2.946	0.014	0.000

**Table 27:** Frequency and current masses for modal analysis accounting for axial forces for the RS case.

Mode	Frequency (Hz)	Current Mass [%]		
	Modal Analysis Accounting for Axial Forces	UX	UY	UZ
1	10.922	5.328	0.009	0.000
2	10.922	0.009	5.332	0.000
3	12.380	0.000	0.000	8.391
4	12.933	0.157	4.744	0.000
5	12.933	4.719	0.153	0.000

**Table 28:** Comparison of MAC values for the first five mode shapes for the RS case.

		Modal Analysis Accounting for Axial Forces				
	MAC	Mode 1	Mode 2	Mode 3	Mode 4	Mode 5
**Modal analysis**	Mode 1	0.9686	0.0005	0.0000	0.0178	0.0051
	Mode 2	0.0005	0.9686	0.0000	0.0053	0.0178
	Mode 3	0.0000	0.0000	0.9296	0.0000	0.0000
	Mode 4	0.0010	0.0601	0.0000	0.9529	0.0018
	Mode 5	0.0600	0.0010	0.0000	0.0019	0.9531

## Data Availability

The original contributions presented in this study are included in the article. Further inquiries can be directed to the corresponding author.

## Appendix A

**Table A1:** Coordinates of the dome nodes.

Node No.	X [m]	Y [m]	Z [m]	Node No.	X [m]	Y [m]	Z [m]
1	10.00	10.00	2.00	26	16.66	10.00	1.13
2	6.68	10.00	1.79	27	16.15	7.45	1.13
3	6.93	11.27	1.79	28	14.71	5.29	1.13
4	7.65	12.35	1.79	29	12.55	3.85	1.13
5	8.73	13.07	1.79	30	10.00	3.34	1.13
6	10.00	13.32	1.79	31	7.45	3.85	1.13
7	11.27	13.07	1.79	32	5.29	5.29	1.13
8	12.35	12.35	1.79	33	3.85	7.45	1.13
9	13.07	11.27	1.79	34	0.0	10.00	0.0
10	13.32	10.00	1.79	35	0.76	13.83	0.0
11	13.07	8.73	1.79	36	2.93	17.07	0.0
12	12.35	7.65	1.79	37	6.17	19.24	0.0
13	11.27	6.93	1.79	38	10.00	20.00	0.0
14	10.00	6.68	1.79	39	13.83	19.24	0.0
15	8.73	6.93	1.79	40	17.07	17.07	0.0
16	7.65	7.65	1.79	41	19.24	13.83	0.0
17	6.93	8.73	1.79	42	20.00	10.00	0.0
18	3.34	10.00	1.13	43	19.24	6.17	0.0
19	3.85	12.55	1.13	44	17.07	2.93	0.0
20	5.29	14.71	1.13	45	13.83	0.76	0.0
21	7.45	16.15	1.13	46	10.00	0.0	0.0
22	10.00	16.66	1.13	47	6.17	0.76	0.0
23	12.55	16.15	1.13	48	2.93	2.93	0.0
24	14.71	14.71	1.13	49	0.76	6.17	0.0
25	16.15	12.55	1.13				
